# Structural basis of odor sensing by insect heteromeric odorant receptors

**DOI:** 10.1126/science.adn6384

**Published:** 2024-06-13

**Authors:** Jiawei Zhao, Andy Q. Chen, Jaewook Ryu, Josefina del Mármol

**Affiliations:** 1 Department of Biological Chemistry and Molecular Pharmacology, Harvard Medical School; Boston, 02115, USA.; 2 Howard Hughes Medical Institute; Boston, 02115, USA.

## Abstract

Most insects, including human-targeting mosquitoes, detect odors through odorant-activated ion channel complexes consisting of a divergent odorant-binding subunit (OR) and a conserved co-receptor subunit (Orco). As a basis for understanding how odorants activate these heteromeric receptors, we report here cryo-EM structures of two different heteromeric odorant receptor complexes containing ORs from disease-vector mosquitos *Aedes aegypti* or *Anopheles gambiae*. These structures reveal an unexpected stoichiometry of one OR to three Orco subunits. Comparison of structures in odorant-bound and unbound states indicates that odorant binding to the sole OR subunit is sufficient to open the channel pore, suggesting a mechanism of OR activation and a conceptual framework for understanding evolution of insect odorant receptor sensitivity.

## One-Sentence Summary

Cryo-EM structures of mosquito odorant receptor complexes reveal an asymmetric architecture.

Animals rely on olfaction for critical behavioral adaptations (1). Mosquitoes, for instance, use their sense of smell to find human hosts and secure a blood meal, and may thereby transmit pathogens like the causative agents of malaria, dengue, and yellow fever (2, 3). In insects such as mosquitoes, hydrophobic volatile compounds are sensed primarily by odorant receptors, a large family of odorant-activated ion channels expressed in the dendritic membrane of olfactory sensory neurons (4–6). Ancestral members of the insect OR family in the order *Archaeognatha* (jumping bristletails), which split off early from the main insect lineage, assemble as homotetrameric ion channels, in which each of the four identical subunits contains a binding pocket that can interact with odorant molecules to elicit channel opening (7–11). Most insects, however, including fruit flies, mosquitoes, ants, and bees, express odorant receptors that assemble as obligate heteromeric complexes formed by two types of subunits: an odorant-binding subunit (OR) and an OR co-receptor called Orco (12–14). Most species contain many divergent ORs, yet express a single Orco (10). The amino-acid sequences of Orcos are highly conserved across a wide range of insect species, and Orcos from different species can functionally substitute for each other (15, 16). Biochemical and electrophysiological studies have established that ORs and Orco assemble as tetramers (14, 17, 18). Current models based on indirect evidence propose an arrangement of two OR and two Orco subunits per complex (13, 19, 20), but this has not been demonstrated. Although Orcos are a necessary part of the odorant receptor complex (21–23), they do not directly interact with any known natural ligands and thus their structural and functional role is unknown.

We report structures of insect heteromeric OR/Orco complexes, in odorant-bound and unbound states, determined by cryogenic electron microscopy (cryo-EM). Because of the public health significance of mosquito olfaction (24), we focused on ORs from mosquito species that are major vectors for yellow and dengue fever (*Aedes aegypti*) and malaria (*Anopheles gambiae*). We determined the structure of a heteromeric complex formed by ORs from these species in complex with a *Apocrypta bakeri* fig wasp Orco that is amenable to structural determination (14, 25). We find that these complexes form asymmetric tetramers containing 1 OR and 3 Orco subunits, where activation of the sole OR subunit upon ligand binding suffices to open the ion conduction pathway. We therefore propose that *in vivo* insect heteromeric receptors could also exhibit a 1:3 stoichiometry, and present structural work on human-sensing mosquito odorant receptors that could aid in the pharmacological targeting of mosquito olfaction to curb the spread of insect-borne diseases.

## The cryo-EM structure of an OR/Orco complex has a 1:3 subunit stoichiometry.

**Figure 1.**
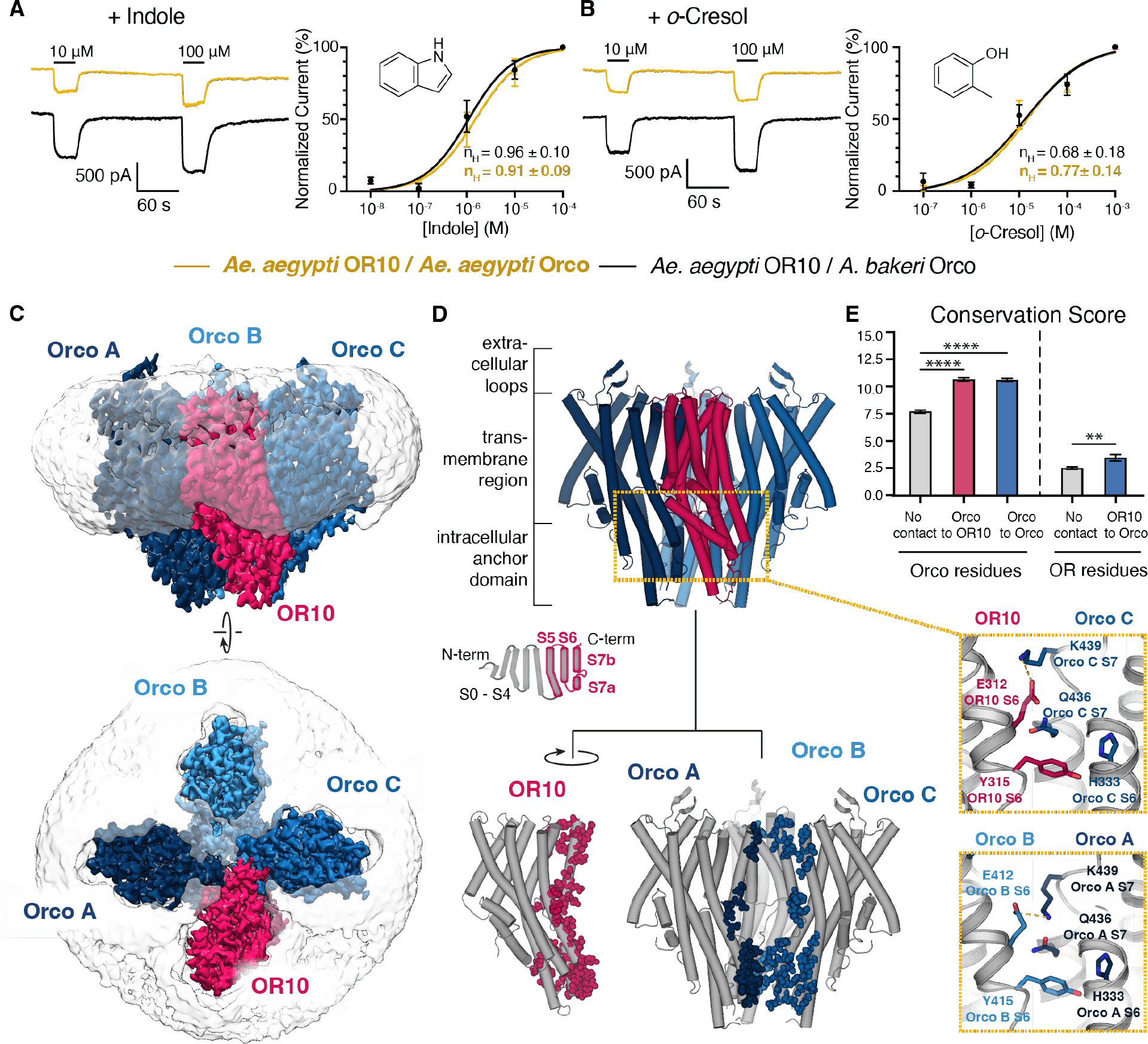
AaOR10/Orco assembles with a 1:3 stoichiometry. **(A)** and **(B)** Comparison of electrophysiological behavior of *Ae. aegypti* OR10 with *A. bakeri* Orco or *Ae. aegypti* Orco. Representative whole-cell currents evoked by indole **(A)** or *o*-cresol **(B)** in HEK293T cells expressing AaOR10 with either *A. bakeri* (black) or *Ae. aegypti* Orco (gold), voltage clamped at 80 mV. The response to *o*-cresol is farther from saturation at high *o*-cresol concentration, limiting the goodness of the fit. Dose-response parameters and statistics can be found in table S2. **(C)** Cryo-EM density map of the AaOR10/Orco complex shown from the side (within the plane of the membrane) and from the top (extracellular surface), colored by proximity to each subunit, as marked. Orcos in shades of blue, OR10 in magenta, detergent micelle in gray. **(D)** Cylindrical helix representation of the complex, indicating overall location of the major features of the complex. Below, all residues found to be involved in contacts between OR10 and Orco subunits are represented as spheres and colored by subunit. OR10 is shown separated and rotated to exhibit the contact residues. Inset: comparison of analogous intersubunit interfaces between OR10 and Orco (top) and between adjacent Orcos (bottom), showing the interactions of a conserved tyrosine (Tyr315 in OR10, Tyr415 in Orco), and also an inter-subunit salt bridge between a conserved lysine (K439 in Orco) and interacting glutamate in OR10 (Glu 312) or Orco (Glu 412). **(E)** Conservation scores (mean +/− SEM) calculated by amino acid position from a sequence alignment of 461 ORs and 47 Orcos across insect species (see Methods). Scale goes from 1 (least conserved) to 11 (identical). Statistical significance assessed using a Mann-Whitney test (**** p<0.0001, ** p=0.0049).

We first focused on the conserved OR10 receptor family, which detects indole and derivatives in various mosquito species (25–27). Co-expression of *Ae. aegypti* OR10 with its conspecific Orco did not yield complexes that were amenable for structural work (fig. S1). We therefore took advantage of the high sequence conservation of Orcos across distant species, a feature that has been widely leveraged to functionally characterize ORs from multiple species (fig. S1, S2). Indeed, Orcos are functionally interchangeable *in vivo* and *in vitro*: previous studies showed that expression of mosquito or moth Orco transgenes rescued neuronal responses to odors in Orco-null *Drosophila* (16). Conversely, *Drosophila* expressing their endogenous Orco are routinely used to heterologously express ORs from distant species for functional characterization (3, 28–31). We therefore co-expressed OR10 from *Ae. aegypti* with a previously structurally characterized Orco from *A. bakeri* to form a complex referred to henceforth as AaOR10/Orco (65% sequence identity and 77% sequence similarity between *Ae. aegypti* Orco and *A. bakeri* Orco, fig. S2). We corroborated the functionality of the AaOR10/Orco complexes by heterologously expressing these complexes in cultured HEK293 cells and performing voltage-clamp electrophysiology to measure their response to indole and *o*-cresol, canonical odorants known to activate AaOR10 heteromers. Consistent with previous studies (11, 14), we found that whole-cell currents of AaOR10/Orco are indistinguishable in EC_50_ and overall dose-response parameters to those obtained with AaOR10 in complex with the Orco from *Ae. aegypti* (fig. 1A, B, Table S2). Therefore, we proceeded with the structural characterization using single-particle cryo-EM of AaOR10/Orco heteromers purified from HEK293 cells (fig. S3).

We transduced HEK 293 cells using a 1:1 ratio of OR to Orco virus, and purified AaOR10/Orco complexes with an affinity tag on the OR subunit. We obtained a map of the AaOR10/Orco complex at 2.9 Å overall resolution enabling unequivocal model building for the majority of the protein (except the flexible intracellular N termini, and portions of the S3-S4, and S4-S5 loops of the Orco subunits) (fig. S3, S4). We found that the heteromeric AaOR10/Orco complexes contain 1 OR subunit and 3 Orco subunits (fig. 1C, D). In order to determine whether the expression level of subunits may dictate the stoichiometry of the resulting complexes, we purified complexes from HEK293 cells transfected with varying ratios of AaOR10 to Orco DNA, and found that increasing the proportion of OR DNA does not lead to the formation of complexes of different biochemical properties, as assessed using Size Exclusion Chromatography and SDS-PAGE (fig. S1 and S3). Subunit assignment to Orco and OR10 was unambiguous, as Orco subunits exhibit strong density for the extracellular loop connecting the S3 and S4 helices (fig. 1C, D, fig. S3, S4, S5). This loop adopts a dramatically distinct conformation in the OR subunit, a difference visible even at early stages of data processing such as *ab-initio* reconstruction. Extensive data processing yielded no evidence of other complex stoichiometries present in the cryo-EM dataset (fig. S5).

Aside from the different extracellular loops, the OR10 and Orco subunits have very similar fold, despite having only 15.1% sequence identity. Each subunit is composed of 7 transmembrane helical segments (S1-S7) and a small re-entrant helix (S0) near the intracellular N-terminal domain (fig. 1D). The overall tetrameric assembly of the AaOR10/Orco heteromer closely resembles that of previously described homotetrameric members of this family, such as the MhraOR5 from the jumping bristletail *Machilis hrabei* (11) and related insect gustatory receptors (32–34). Most of the protein is embedded within the membrane plane, and a small ‘anchor’ domain protrudes into the intracellular space (fig. 1C, D). The anchor domain, named for its role in anchoring subunits together through a tight network of intersubunit interactions, is formed by the coalescence of the intracellular portions of the helices S5, S6 and S7a from all subunits. Orco is conserved across insects yet can form functional complexes with many divergent ORs. We used PDBePISA to analyze the intersubunit interactions (35) and found that they all involve the carboxy-terminal portion of the protomers, corresponding to the S5, S6 and S7 helices, namely the anchor domain and pore region (fig. 1D and fig. S2). We mapped the residues connecting OR10 and Orco onto an alignment of Orcos and ORs constructed using 47 Orcos and 461 ORs from five distant species (fig. S2). We used JalView (36) to calculate the conservation score per amino-acid position, a metric that reflects the conservation of physicochemical features at each position in the alignment.

We found that the amino-acid positions at the contact interface between OR10 and Orco subunits have higher conservation scores than the remainder of the protomers (fig. 1E). The residue with the highest conservation score in ORs corresponds to Tyr315 in AaOR10 (a score of 9 for Tyr315, compared to a 2.5 average score for the rest of the OR positions). In the structure, Tyr315 protrudes deeply into the anchor domain of the neighboring Orco subunit, tightly interacting with a pocket of conserved Orco residues (fig. 1D, inset). All intersubunit interfaces are dominated by van der Waals interactions; however, each Orco-Orco interface also contains 2 salt bridges, while OR10 only establishes a salt bridge with one of its neighboring Orcos (fig. 1D, inset). Importantly, the residues in Orco that are in direct contact with AaOR10 in our structure exhibit 97% sequence identity and 100% sequence similarity to those in *Ae. aegypti* Orco, providing a structural explanation to the ability of Orcos to substitute for each other.

## A conformational change in the OR subunit alone is sufficient to open the pore of the AaOR10/Orco complex.

Thus far, the only structural insight into gating mechanisms of insect ORs comes from the cryo-EM structure of the homotetrameric MhOR5 from the jumping bristletail (11). In MhOR5, there are no Orco subunits and all four identical OR subunits contain an odorant binding pocket and contribute their S7b helix to the pore of the ion conduction pathway. In the heteromeric OR/Orco complexes, however, although both Orco and OR subunits interface the pore, only OR subunits have odorant binding pockets. As a result, two mechanisms of activation are possible: odorant binding to the OR subunit(s) could elicit a global conformational change involving movement of both OR and Orco pore helices, resulting in a radial dilation of the pore. Alternatively, channel activation could result solely from movement of the OR subunit(s), with the Orco subunits remaining static.

**Figure 2.**
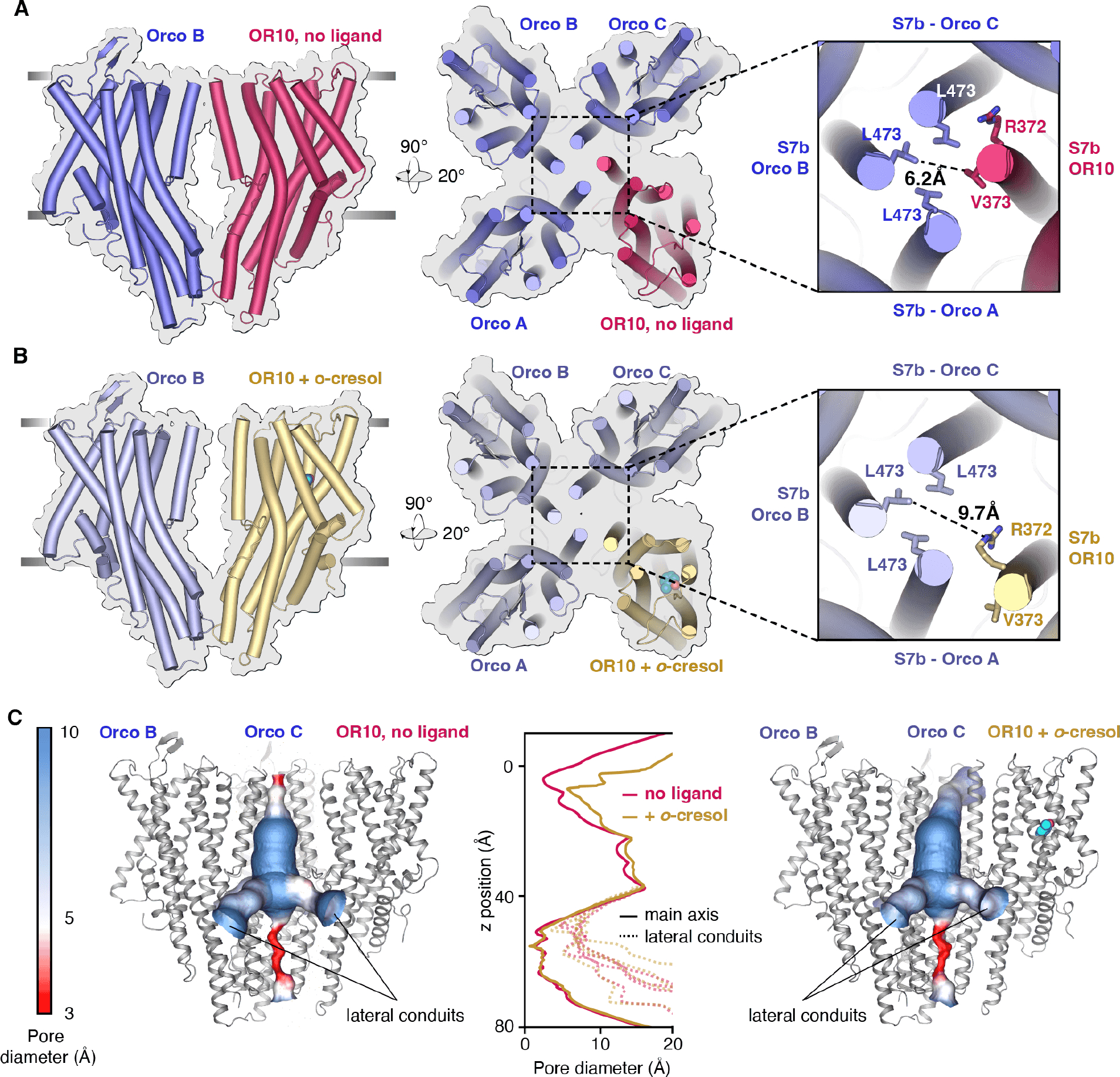
The AaOR10/Orco channel gates through outward displacement of the OR10 pore helix. **(A)** and **(B)** Views of the AaOR10/Orco complex in the unbound **(A)** and *o*-cresol-bound **(B)** structures. Leftmost: Lateral view of the OR subunit and the diagonal Orco subunit. Right: Top view of the complexes, with a close-up view of the residues facing the lumen of the pore at the gate. Measurement of distances between the Orco and OR pore residues in the unbound **(A)** and *o*-cresol-bound **(B)** structures is taken from center atoms, using PyMOL. **(C)** The ion permeation pathways of the unbound (left) and *o*-cresol bound (right) structures, colored by pore diameter. The front Orco A subunit is not shown to permit visualization of the cavity. The center vestibule of the ion conduction pathway is continuous with four lateral conduits that allow ion permeation; two are shown in each structure. The intracellular ‘anchor’ domain remains closed in unbound and *o*-cresol bound structures. The plot shows the diameter of the ion permeation pathway, with respect to distance from the outer membrane boundary towards the intracellular space, in Å. The diameter of the impermeable central path through the anchor domain is shown in solid lines, while those of the lateral conduits are in dashed lines.

We therefore determined the cryo-EM structure of the AaOR10/Orco complex bound to *o*-cresol to an overall resolution of 2.9 Å. We found a pronounced lateralized dilation of the channel pore, elicited by the outward movement of the OR10 S7b pore helix (fig. 2A, B). The OR10 S7b helix shifts diagonally outwards from the main channel axis, increasing the distance to the opposing Orco subunit from 6.2 Å to 9.7 Å. The resulting open pore is likely wide enough to be permeable to hydrated cations, a result consistent with electrophysiological data showing that the channel, like other insect OR/Orco complexes, passes both monovalent and divalent cations (fig. S7, Table S3). From this extracellular gate, the channel continues into a wide vestibule lined with hydrophilic residues in the transmembrane portion of the complex (fig. 2C). At the base of this vestibule, ions flow through four lateral conduits into the intracellular space in a quadrivial pore architecture (fig. 2C), analogous to what has been observed in structures of homotetrameric members of this family (11, 14, 32–34).

A comparison of the ion conduction pathway of the odorant bound and unbound structures shows that the major displacement of the OR pore helix at the extracellular gate is the only conformational change along the pore, with the remainder of the ion conduction path nearly unchanged (fig. 2A, B, C and fig. S8). The Orco subunits in the heteromer remain virtually static during channel activation, with a RMSD of 0.9 Å between the liganded and unliganded structures (fig. S8), compared to an overall RMSD of 2.4 Å for the OR10 subunit. The lack of cooperative movement of Orco subunits in response to odorant binding agrees with the Hill coefficient of one estimated from dose-response activation curves (fig. 1A, B). Therefore, the presence of the OR subunit is sufficient to impart odorant specificity to the complex and lead to channel opening without any concerted movement of the other subunits.

The local resolutions of the odorant bound and unbound structures further support this conclusion. In both odorant-bound and unbound structures of the heteromeric complex, the density corresponding to the Orco subunits is overall stronger than that of the OR subunit, which suggests a higher conformational flexibility localized to the OR subunit (fig. S4).

## Mechanism of AaOR10/Orco activation by odorant.

**Figure 3.**
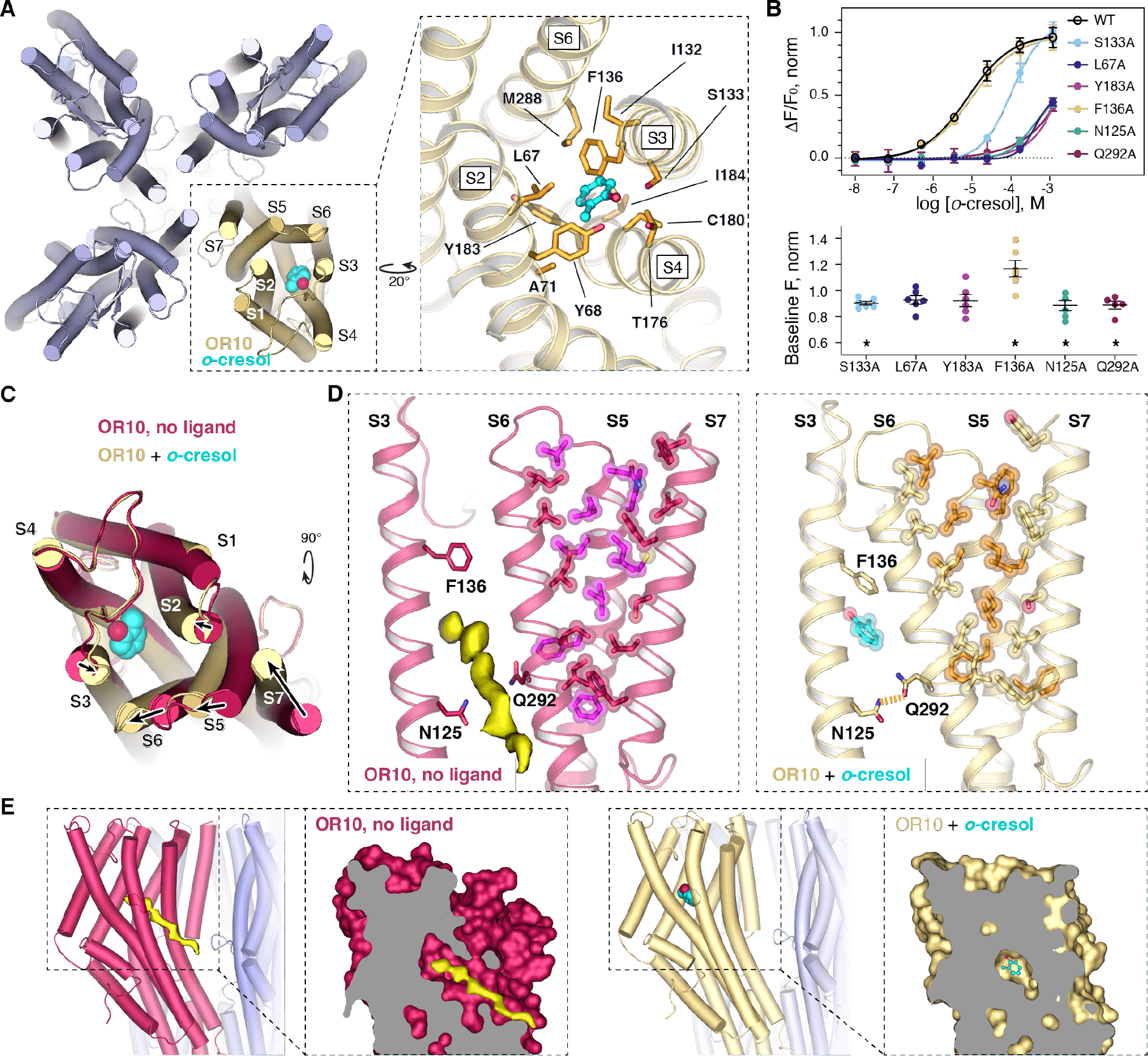
Odorant binding and mechanism of activation of AaOR10/Orco. **(A)** Top view of the *o*-cresol-bound AaOR10/Orco complex. Inset: close-up of the ligand-binding pocket showing the *o*-cresol-interacting residues. **(B)** Top, *o*-Cresol dose-response curves of AaOR10/Orco mutants. Bottom, baseline fluorescence of the mutants, all normalized to the baseline of wild-type AaOR10/Orco on the same plate in order to account for inter-plate variation. Experimental details and curve parameters (including N and EC50) in supplementary table 4 and methods. **(C)** Overlay of the unbound and *o*-cresol bound structures showing a top view of the AaOR10 subunit, displaying the rearrangements of helices in the presence of *o*-cresol. **(D)** Close-up of helices S3, S6, S5, and S7 in the unbound (left) and *o*-cresol-bound (right) structures. Interacting residues in S5, S6, and S7 within 5 Å from each other are shown in ball-and-stick representation. Note the unmodeled density in the unbound state wedged between helices S3-S6 and partially occupying the pocket. **(E)** Lateral views of the complex in unbound (left) and *o*-cresol bound (right) states, displaying the unmodeled density found in the unbound state. Insets: close-ups of the unmodeled density (yellow surface), *o*-cresol (cyan sticks), and the ligand-binding pocket in AaOR10, which is shown as a clipped surface. The unbound state exhibits a continuous tunnel connecting the ligand-binding pocket to the membrane, where we find the unmodeled density. When bound with *o*-cresol, the tunnel is closed by the coming together of S3 and S6 helices.

In the odorant-bound structure, we observed clear density for *o*-cresol in the transmembrane portion of the OR10 subunit, ~18 Å deep from the extracellular space, in a pocket formed by the coalescence of the S2, S3, S4 and S6 helices (fig. 3A, fig. S9A). We used molecular docking to assess the pose of the ligand (37). All top binding poses fit well within the observed density (fig. S9B, C), and suggest that *o*-cresol binding is mediated by hydrophobic and aromatic residues such as Tyr183, Leu 67, and by a hydrogen bond formed between the hydroxyl groups of the *o*-cresol and Ser133. Mutation of these residues to alanine decreases the apparent affinity of *o*-cresol activation, seen as a right-shift in the dose-response curve assessed using an established calcium flux functional assay (11, 14) (Fig 3B, Table S4). The position of the AaOR10 binding pocket closely resembles those of the homotetrameric MhOR5 (fig. S10).

How does odorant binding lead to receptor activation? Comparison of the unbound and *o*-cresol bound structures suggests that the shift in position of the OR10 S7b pore helix gates the ion conduction pathway and hence determines receptor activation (fig. 2B and fig. 3B). That shift is tightly coupled to a displacement of the adjacent helix S5, which in turn is coupled to helix S6 through extensive van der Waals interactions (fig. 3A, B and fig. S8). Helix S6 thus links the odorant binding pocket with the S7b pore helix.

The cryo-EM dataset of the unbound AaOR10/Orco complex contained two distinct subsets of particles. The largest classes contained particles in the inactive conformation, with the pore helix S7b in the ‘closed’ state, as expected (fig. S4A, classes 0, 2 and 3). A smaller but unambiguous class was in the active conformation, even in the absence of odorant, with the S7b pore helix in the ‘open’ state (fig. S4A, class 1). The existence of active and inactive states in the unbound dataset suggests that even in the absence of odorant, AaOR10/Orco is an equilibrium mixture, consistent with the well-characterized high baseline activity of insect odorant receptors in the absence of odorant ligands (38).

The density map for the unbound, inactive conformation contains an elongated feature protruding from the membrane into the odorant binding pocket of the OR subunit (fig. 3D, E). The strength and shape of this unmodeled density suggest that it represents a lipid molecule co-purified with the heteromeric complex. One end of this density partially occludes the odorant binding pocket (fig. 3C). The long tail of this density, in turn, is wedged into the groove between the S3-S6 and extends into the transmembrane space, helping stabilize the inactive conformation by preventing helix S6 from moving towards helix S3. The side chain of Phe136 in S3 also impedes a shift of helix S6 (fig. 3D) away from its position in the inactive conformation, through a contact with Tyr285 in helix S6. Thus, both the presumptive lipid and the phenyl ring of Phe136 appear to stabilize the inactive conformation of AaOR10/Orco complex by holding S6 in place. Mutation of Phe136 to alanine significantly increases the baseline activity of the receptor, likely shifting the conformational equilibrium towards the active state, consistent with its role in stabilizing the closed conformation (fig. 3B, Table S4).

In the *o*-cresol bound structure, density corresponding to the hypothetical lipid is absent. Instead, density of the *o*-cresol molecule occupies the odorant binding pocket (fig. 3D, fig. S9). Ejection of the lipid and rotation of Phe136 away from the S6 together clear the space between helices S6 and S3 (fig. 3C, D), allowing helix S6 to move towards helix S3 and give rise to the active receptor conformation and opening of the channel pore. Closure of the S3-S6 gap also allows a hydrogen bond to form between Gln292 in S6 and Asn125 in S3 (fig. 3D), further stabilizing the active conformation. Indeed, mutation of either interacting partner of this hydrogen bond between S3 and S6 decreases channel activation (fig. 3B).

In summary, by destabilizing the inactive conformation and stabilizing the active conformation, *o-*cresol shifts the receptor conformational equilibrium towards the active state, resulting in pore opening.

## A different heteromeric OR/Orco complex also has a 1:3 subunit stoichiometry and a similar mode of channel activation.

**Figure 4.**
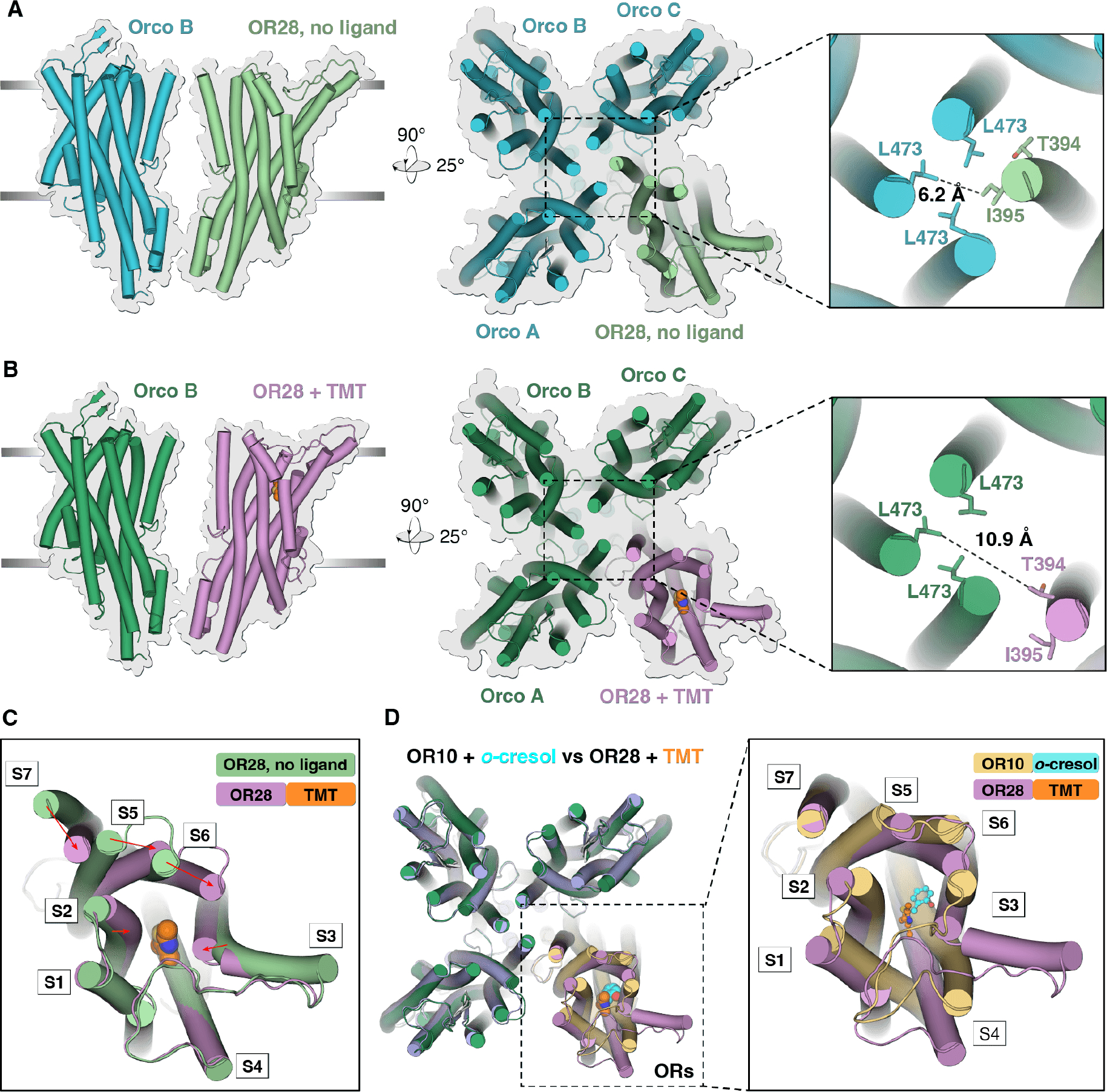
Cryo-EM structures of an OR/Orco complex from the mosquito *Anopheles gambiae*, in unbound and odorant-bound states. **(A)** and **(B)** Views of the AgOR28/Orco complex in the unbound state **(A)** and bound to 2,4,5-trimethylthiazole (TMT) **(B)**. Leftmost: Lateral view of the OR subunit and the diagonal Orco subunit. Right: Top view of the complexes, with a close-up view of the residues facing the lumen of the pore at the gate. Measurement of distances between the Orco and OR pore residues in the unbound **(A)** and TMT-bound **(B)** structures is taken from center atoms, using PyMOL. **(C)** Top view of the AgOR28 subunit, displaying the rearrangements of helices in the presence of TMT, in a pattern closely resembling that of the AaOR10 complex (see fig. 3). **(D)** Left, overlay of top views of ligand-bound structures of AaOR10/Orco and AgOR28/Orco. Right inset, overlaid top views of the *o*-cresol-bound AaOR10 and TMT-bound AgOR28 subunits.

To determine if the 1:3 subunit stoichiometry can be observed with other members of the OR family, we turned to studying the OR28 receptor of *An. gambiae*, a main vector of malaria, that responds to the natural odorants acetophenone (a plant volatile) and 2,4,5-trimethylthiazole (henceforth TMT; an animal odor, originally isolated from anal secretions of mammals) (39, 40). We confirmed through electrophysiological recording that AgOR28 co-expressed heterologously with *A. bakeri* Orco assembled into TMT-responsive complexes (fig. S11). We then determined the TMT-bound and unbound structures of AgOR28 in complex with *A. bakeri* Orco, to an overall resolution of 2.95 Å and 2.62 Å, respectively (fig. 4 A, B, C, fig. S11, S12, S13).

The AgOR28/Orco structures had the same 1:3 subunit ratio as did the AaOR10/Orco complex (fig. 4) and a lateralized pore opening, elicited by a conformational change in the OR subunit only (fig. 4B, C and fig. S14). We did not detect any other stoichiometries of AgOR28/Orco throughout the course of data processing (fig. S12). PDBePISA analysis of the intersubunit interfaces in the AgOR28/Orco complex revealed that the majority of the interactions between AgOR28 and Orco are mediated by the same Orco residues that mediate interactions with the AaOR10 subunit (fig. S2). Furthermore, the position of the three Orco subunits in complex with either AaOR10 and AgOR28 is nearly identical (fig. 4D), illustrating modular assembly of a single Orco with many ORs.

As was the case in the OR10 complex, the local resolution of the OR28 subunit in both bound and unbound structures is lower than that of Orco, suggesting a higher conformational flexibility in the OR28 subunit than the Orco subunits (fig. S12). The cryo-EM dataset of the unbound structure also contains an active state, suggesting that, like the AaOR10/Orco complex, the AgOR28/Orco complex exists in an equilibrium between states in the absence of odorant.

Clear density for TMT was found in a binding pocket roughly in the same position as the AaOR10 binding pocket (fig. 4 C and D and fig. S14). Molecular docking of TMT into the AgOR28/Orco complex placed the top poses well within the experimental ligand density, and suggested that TMT binding is largely mediated by hydrophobic interactions (fig. S14).

Overall, subunit stoichiometry, mechanism of pore opening and ligand binding of the AgOR28/Orco complex largely resembles the observations of the AaOR10/Orco complex, suggesting that these might be conserved features of this family of proteins.

## Discussion.

Here we report cryo-EM structures of two distinct heteromeric OR/Orco complexes, both in odorant-bound and unbound states. We consistently observe a stoichiometry of 1 ligand-binding OR to 3 Orcos in these structures. Based on these *in vitro* structures, we suggest that insect heteromeric complexes could also contain a single OR subunit per complex *in vivo*. In this proposed stoichiometry, odorant binding to the single OR binding site is sufficient to open the pore. Orco subunits, which line the majority of the ion conduction pathway, remain static throughout receptor activation. Their role, therefore, is to provide a structurally sound scaffold onto which ORs can assemble to gate the pore, conferring diversified odorant sensitivity.

Asymmetric heteromeric ion channel assemblies are common in nature. For instance, nicotinic acetylcholine receptors (41), GABA_a_ receptors (42), cyclic nucleotide-gated channels (43) and ionotropic glycine receptors (44–46) can contain heterogeneous subunits that assemble in different stoichiometries depending on the tissue. Recent structural studies of these heteromeric complexes show that pore opening also occurs with striking asymmetry (47, 48). It is likely that the recent advances in cryo-EM structure determination allow us to now determine higher resolution structures of heteromeric complexes without the need to impose symmetry during data processing, unveiling previously unappreciated complexity in the gating mechanisms of asymmetric assemblies.

Recent comparative studies of insect olfaction showed that odorant receptors of earlier derived insect species, such as jumping bristletails, operate as homotetramers. Homotetrameric members of the family of insect gustatory receptors (from which the OR family derived) seem to require more than 1 subunit to elicit channel activation, as evidenced by their Hill coefficients of ~2.5 in heterologous studies (33). In contrast, all recently derived insect clades have adopted a heteromeric OR/Orco model, suggesting an adaptive advantage over the homomeric ORs (8, 9, 49). One possibility is that the reduction in number of odorant binding subunits increases odorant sensitivity of the complexes. This sensitivity addresses a conserved challenge of olfactory systems, that need to detect trace amounts of poorly soluble odorant stimuli. In most vertebrate odorant receptors, which belong to the G-protein coupled receptor family, the binding of a single odorant molecule suffices to initiate neuronal signaling (50). With a proposed 1:3 subunit stoichiometry, insect odorant receptors would also have evolved along a similar path, resulting in a single odorant molecule per signaling event.

Olfactory systems, whether invertebrate or vertebrate, often include a large and diversified repertoire of receptors to collectively tile the ethologically relevant chemical space and discriminate molecules with requisite specificities (1). Within insects, *Drosophila melanogaster* has 62 different ORs (51), *Ae. aegypti* has 117 (52), and species in the *Formicidae* (ant) family can have over 400 (53, 54). A heteromeric assembly may allow new ORs to quickly evolve through binding pocket and pore mutations yet still easily assemble onto an invariant scaffold of Orco, rather than having to additionally evolve novel multimerization ‘anchor’ domains or cooperative mechanisms of gating through multiple subunits. This adaptation efficiently enables a massive and rapid diversification of ORs in recently diverged insect species, allowing them to sense and discriminate a broad swath of odorants.

The robustness of formation of the 1:3 stoichiometry described in this work suggests that it might represent a physiologically relevant state. If other subunit stoichiometries are in fact the predominant functional form in native olfactory sensory neurons, additional regulatory mechanisms could be in place *in vivo* to bias the formation of alternative complexes instead of the 1:3 complex described in this manuscript. In either case, intense selective pressure over millions of years resulted in the heteromeric insect odorant receptor family, and enabled the exceptional olfactory adaptations of insects that allowed them to colonize myriad ecological niches. How these complexes form, assemble, and gate in response to odors remains an outstanding question for future investigation. Our findings, with an unexpected subunit stoichiometry, propose a new model to conceptualize odorant binding and impact our thinking of how odor processing takes place in the sensory neurons of insects.

## Supplementary Material

Methods, Supp Figures and Tables

MDAR

## Data Availability

The cryo-EM density maps and the atomic models of the complexes have been deposited in the Electron Microscopy Data Bank and Protein Data Bank with the following accession numbers EMD-42848 PDB:8V00 (AaOR10/Orco, apo), EMD-42850 PDB:8V02 (AaOR10/Orco, *o*-cresol-bound), EMD-42945 PDB:8V3C (AgOR28/Orco, apo), EMD-42946 PDB:8V3D (AgOR28/Orco, 2,4,5-trimethylthiazole-bound). All other data needed to evaluate the conclusions in the paper are present in the paper and/or the Supplementary Materials.
