# Supplementary material for: Structural basis of odor sensing by insect heteromeric odorant receptors
Source: Science. Author manuscript; Available in PMC 2024 Jul 10. (PMC11235583; doi:10.1126/science.adn6384)
Supplement: Methods, Supp Figures and Tables [file NIHMS2003466-supplement-Methods__Supp_Figures_and_Tables.pdf]

Supplementary Materials:

Title: Structural basis of odor sensing by insect heteromeric odorant receptors.

Authors: Jiawei Zhao[1], Andy Q. Chen[1], Jaewook Ryu[1] and Josefina del Mármol[1,2,*]

Affiliations:

[1] Department of Biological Chemistry and Molecular Pharmacology, Harvard Medical School; Boston, 02115, USA.

[2] Howard Hughes Medical Institute; Boston, 02115, USA.

[*] Correspondence: josefina_delmarmol@hms.harvard.edu

Materials and Methods.

Expression and purification of AaOR10/Orco and AgOR28/Orco complex.

The coding sequences of AbOrco and AgOR28 were a gift from the lab of Vanessa Ruta at the Rockefeller University, used in Butterwick et al, 2018 (*14*). The coding sequence of AaOR10 was codon optimized and synthesized (Twist Bioscience). Gene fragments of full-length AaOR10 and AgOR28 were separately cloned into a pEG BacMam vector (*55*) containing an N-terminal super-folder GFP and an HRV 3C protease site. The gene fragment of full length AbOrco was cloned into a pEG BacMam vector containing an N-terminal mCherry and an HRV 3C protease site. Sf9 insect cells (Expression Systems) were used to produce baculoviruses, each containing one construct. HEK293S GnTI⁻ cells (ATCC CRL-3022) were grown at 37 °C with 8% carbon dioxide in Freestyle 293 medium (Gibco) supplemented with 2% (v/v) fetal bovine serum (Gibco). Cells were grown to 3×10^6 cells/ml and transduced with baculoviruses for protein expression. To co-express OR and Orco, baculovirus containing OR or Orco constructs was added in 1:1 MOI ratio to HEK293S GnTI⁻ cells, to a total volume of baculoviruses of 1/10 of the HEK293S GnTI⁻ cell culture. After 24 hours, 10 mM sodium butyrate (Sigma-Aldrich) and 0.36% glucose (Sigma-Aldrich) were added to the cells, and the temperature was dropped from 37 °C to 30 °C for the remainder of the incubation. Cells were collected by centrifugation 48 hours post-transduction and stored at −80 °C until the day of purification.

For purification, cell pellets were thawed in room-temperature water, and resuspended in 100 mL of solubilization buffer per liter of cell culture. The solubilization buffer was composed of 20 mM HEPES/NaOH (pH 7.5), 150 mM NaCl, 0.5% (w/v) Lauryl Maltose Neopentyl Glycol (LMNG; Anatrace), 0.1% (w/v) cholesterol hemisuccinate (CHS; Sigma-Aldrich), 2.5 µg/mL leupeptin, and 160 µg/mL benzamidine. The cell pellets in the solubilization buffer were homogenized with a Dounce homogenizer in an ice-water bath and stirred with a magnetic stir bar in the cold room for an hour. The mixture was clarified by centrifugation at 90,000 g for 25 minutes, and the supernatant was added to 1 mL of anti-GFP nanobody-coupled Sepharose resin (*56*) per liter of cell culture. After washes with 20 mM HEPES pH 7.5, 150 mM NaCl, 0.01% LMNG, and 0.002% CHS, the OR/Orco complex was eluted by mixing 50 µg of 3C protease (Sigma-Aldrich) with every 1 mL of resin and gentle rotation at 4 °C for an hour. The sample was then concentrated and injected into a Superose 6 Increase column (Cytiva) pre-equilibrated with 20 mM HEPES pH 7.5, 150 mM NaCl, 0.002% LMNG, 0.0004% CHS. Peak fractions containing OR/Orco complex were

pooled and concentrated to A280 = 4.7 (the OR10/Orco sample) or 1.8 (the OR28/Orco sample). The proteins were used immediately for cryo-EM grid preparation, or flash-frozen and kept at -80 °C until use. A single freeze-thaw cycle does not significantly affect the protein behavior on grids. To prepare ligand-bound samples, *o*-cresol was added to the OR10/Orco sample to a final concentration of 1 mM, and 2,4,5-trimethylthiazole was added to the OR28/Orco sample to a final concentration of 5 mM. The ligands were incubated with samples at room temperature for 1 hour before making grids.

Cryo-EM sample preparation and data acquisition. Cryo-EM grids were frozen using a Vitrobot Mark IV (FEI) as follows: 3 μL of the concentrated sample was applied to a glow-discharged Quantifoil R1.2/1.3 holey carbon 400 mesh gold grid, blotted for 5 s in 100% humidity at 4 °C, and plunge frozen in liquid ethane cooled by liquid nitrogen. Cryo-EM data were recorded on a Titan Krios (FEI) operated at 300 kV, equipped with a Gatan BioQuantum GIF/ K3 direct electron detection camera. SerialEM was used for automated data collection. The defocus range is -1.0 to -2.0 μm. Movies of the OR10 sample without a ligand were collected at a magnification of 105,000x in super-resolution mode with a physical pixel size of 0.827 Å/pixel. Part of the data was collected with a 30° tilted stage. 60 frames were collected with a total dose of 60 electrons per Å^2. Movies of the OR10 sample with *o*-cresol ligand were from 2 data collection sessions, both at a magnification of 105,000x in counting mode. The first session used a physical pixel size of 0.825 Å/pixel, and 58 frames were collected with a total dose of 55 electrons per Å^2. The second session used a physical pixel size of 0.83 Å/pixel, and 64 frames were collected with a total dose of 58 electrons per Å^2. Part of the data in the second session was collected with a 30° tilted stage. Movies of the OR28 sample without a ligand were collected at a magnification of 81,000x in super-resolution mode with a physical pixel size of 1.061 Å/pixel. Part of the data was collected with a 30° tilted stage. 50 frames were collected with a total dose of 50 electrons per Å^2. Movies of the 2,4,5-trimethylthiazole-bound OR28 were collected at a magnification of 81,000x in super-resolution mode with a physical pixel size of 1.061 Å/pixel. Part of the data was collected with a 30° tilted stage. 50 frames were collected with a total dose of 50 electrons per Å2.

Cryo-EM data processing. Cryo-EM data was processed using CryoSPARC Live and CryoSPARC (*57*). Motion correction of movies, contrast transfer function estimation, particle picking, particle extraction with 2-fold binning, and the first round of 2D classification were carried out in CryoSPARC live. For movies collected in super-resolution mode, micrographs were binned over 2x2 pixels after motion correction in CryoSPARC Live. Multiple rounds of 2D classification were run in CryoSPARC to enrich tetramer particles. Selected good particles from 2D classification were used for creating initial maps using the Ab-Initio reconstruction job. Two to four initial maps were used as references in the following rounds of heterogeneous refinement jobs, which effectively removed junk particles. After 2D classifications and multiple rounds of heterogeneous refinement, a "seed" method was used to enrich good particles from the raw particles (*58*). Briefly, raw particles were split into a few subgroups, and each subgroup was combined with the "seed" particles that were selected from previous 2D classification and heterogeneous refinement. Heterogeneous refinement was then carried out for each combined subgroup. The good classes from each heterogeneous refinement job were combined, and redundant particles were removed by CryoSPARC automatically. After a few rounds of seed-facilitated heterogeneous refinement and non-uniform refinement, 3D classification jobs were carried out to separate particles belonging to active or inactive states, using a mask focusing on the OR subunit. After non-uniform refinement, good particles from the 3D classification were re-

extracted. Non-uniform refinement, local CTF refinement, heterogeneous refinement, and local refinement were used to further process particles until a high-quality map was generated.

OR10/Orco without ligand. 11,377 movies were collected for the OR10/Orco sample without a ligand. The 3D classification job generated four classes, one active and three inactive classes. After further processing, only one of the three inactive classes generated a high-quality map with an overall resolution of 2.88 Å as estimated by CryoSPARC with a cutoff for the Fourier shell correlation (FSC) of 0.143.

OR10/Orco with o-cresol. 5,463 and 5,374 movies were collected in two sessions for the OR10/Orco sample with *o*-cresol. 3D classification jobs of each dataset showed 2 classes in inactive and active state. Particles that belong to the active state were re-extracted and further processed with jobs of non-uniform refinement, local CTF refinement, heterogeneous refinement, 3D classification, and local refinement. Good particles from two datasets were merged. To merge two datasets with different pixel sizes (0.825 Å/pixel in dataset 1, 0.83 Å/pixel in dataset 2), particles in the first dataset were re-extracted with a box of 332 pixels, and Fourier cropped to 330 pixels. Particles in the second dataset were re-extracted with a box of 330 pixels and merged with the first dataset. Merged particles were further processed with non-uniform refinement and local refinement, and generated a map with an overall resolution of 2.9 Å.

OR28/Orco without ligand. 10,253 movies were collected for the OR28 sample without a ligand. The focused 3D classification job with a mask on the OR28 subunit generated three inactive classes and one active class. Further processing one of the inactive classes generated a map with an overall resolution of 2.62Å.

OR28/Orco with 2,4,5-trimethylthiazole. 5,690 movies were collected for the OR28 sample with 2,4,5-trimethylthiazole ligand. The 3D classification job generated two active classes and an inactive class. Further processing one of the active classes generated a map with an overall resolution of 2.95 Å.

Model Building. The published structure of *A. bakeri* Orco (pdb 6c70) and the Alpha Fold v2 model of AaOR10 or AgOR28 was used as a starting point for manual model building in Coot (*59*). The density maps were of sufficient quality for building the majority of the protein, specifically, the following residues were built:

AaOR10/Orco, apo state:
- Orco subunits: 4-159, 168-243, 313-474
- OR10 subunit: 1-375 (all)

AaOR10/Orco, *o*-cresol-bound state:
- Orco subunits: 7-159, 168-243, 313-474
- OR10 subunit: 1-375 (all)

AgOR28/Orco, apo state:
- Orco subunits: 4-159, 168-243, 313-474
- OR28 subunit: 3-397

AgOR28/Orco, TMT-bound state:
- Orco subunits: 4-159, 168-243, 313-474
- OR28 subunit: 2-397

The models were refined using real space refinement implemented in PHENIX (*60*) for 5 macro-cycles with secondary structure restraints applied and without symmetry enforced. The *o*-cresol-bound and 2,4,5-trimethylthiazole bound models were refined including the ligands, with restraints obtained using eLBOW implemented in Phenix. The ligand pose was obtained by docking each ligand into the pocket using Glide implemented in Maestro (Schrödinger) and selecting the top pose by docking score, which in both cases (*o*-cresol in AaOR10 and TMT in AgOR28) corresponded to the best fit with the observed cryo-EMdensity (see docking details below). Model statistics were obtained using MolProbity. Images of the maps were created using UCSF ChimeraX (*61*). Images of the model were created using PyMOL and UCSF ChimeraX.

Docking. *O*-cresol and TMT were docked using Glide implemented in Maestro (Schrödinger) (*37*). The experimental structures the ligand bound AaOR10/Orco and AgOR28/Orco were prepared for docking within Maestro, and a 20 $Å^3$ cubical grid search was selected with a center of mass placed in the binding pocket. *O*-cresol and TMT structures were prepared using Epik52 in Maestro to generate their possible tautomeric and ionization states, all optimized at pH 7.4 ± 2. All ligands were docked within the grid, and the top 5 poses are presented in Figure S9 and Figure S14. For each structure, the top pose was selected for model building and refinement.

Electrophysiology. HEK293S GnTI$^-$ cells (ATCC CRL-3022) were maintained in suspension in Freestyle media supplemented with 10% (v/v) fetal bovine serum (FBS) at 37 °C with 5% (v/v) carbon dioxide. Cells were plated and infected 24–48 h before recording with the same pEG BacMam GFP and mCherry-tagged AaOR10/Orco construct used for structure determination. Electrodes were drawn from borosilicate patch glass (Sutter Instruments) and polished (MF-83, Narishige Co.) to a resistance of 3–6 MΩ when filled with pipette solution. Analogue signals were digitized at 20 kHz (Digidata 1550B, Molecular Devices) and filtered at 1 kHz using the built-in four-pole Bessel filter of a Multiclamp 200B patch-clamp amplifier (Molecular Devices) in whole-cell or patch mode. Whole-cell recordings were baseline-subtracted offline.

Whole-cell recordings were performed using an extracellular (bath) solution composed of 150 mM NaCl, 2 mM CaCl2, 10 mM glucose, 10 mM HEPES-Na/HCl (pH 7.4, 310 mOsm/kg) and an intracellular (pipette) solution composed of 150 mM KCl, 10 mM NaCl, 5 mM EGTA-K, 10 mM HEPES-K/HCl (pH 7.4, 310 mOsm/kg). Stock *o*-cresol, indole, and 2,4,5-trimethylthiazole solution was prepared by dissolving in DMSO at 1 M, and working solutions were prepared by diluting stocks in extracellular solution. The DMSO concentration was normalized across solutions, at .1%. Solutions were locally perfused using a microperfusion system (ALA Scientific Instruments).

For the ion-selectivity studies the intracellular (pipette) solution consisted of 150 mM CsCl, 10 mM HEPES-Cs (pH 7.4, 290mOsm/kg). The extracellular solutions for monovalent cations (X = Na, K) consisted of 150 mM XCl, 10 mM HEPES-X/HCl (pH 7.4, 290mOsm/kg). The extracellular solutions for divalent cations (X = Ca, Mg) consisted of 110 mM XCl2 10 mM HEPES-Cs/HCl (pH 7.4, 290mOsm/kg). *O*-cresol was diluted to 1mM in extracellular solution. Solutions were perfused locally in a bath composed of 150 mM NaCl, 10 mM HEPES-Na/HCl (pH 7.3, 290mOsm/kg). Liquid junction potentials were calculated with the built-in pCLAMP JPCalcW program. Erev was obtained by linear interpolation of steady-state currents measured around the reversal, as performed in (Butterwick et al.)

Cell-based GCaMP fluorescence calcium flux assay. This assay was performed in a similar manner to (Butterwick) (del Marmol). All DNA constructs used in this assay were cloned into a modified pME18 s vector that contains a SV40 promoter and no fluorescent marker, flanked by AscI/NotI restriction enzyme sites for efficient cloning. Each transfection condition, sufficient for 2 × 8 wells in a 96-well plate (Greiner Bio-One), contained 640 ng each of plasmid encoding GCaMP6s (Addgene #40753), AbakOrco, and AaegOR10 or its mutants. This yields a total of 1.92 µg of DNA per condition, diluted in 80 µL of OptiMem with 3.68 µL of P3000 reagent (Invitrogen). This was mixed with another 80 µL of OptiMem with 3.68 µL of Lipofectamine 3000 reagent (Invitrogen), and incubated for 30 minutes at room temperature.

HEK293 cells were maintained in high-glucose DMEM supplemented with 10% (v/v) FBS and 1% (v/v) GlutaMAX at 37 °C with 5% (v/v) carbon dioxide. Cells were detached using trypsin and resuspended in FluoroBrite DMEM (Gibco) supplemented with 10% (v/v) FBS to a final concentration of 1 × 106 cells/ml. 1.44 mL of cells were added to each transfection condition, mixed, and 90 µL added to each well on the plate. After 18-20 hours of incubation at 37 °C, this medium was replaced with 80 µl reading buffer (20 mM HEPES/NaOH (pH 7.4), 1× HBSS (Gibco), 3 mM Na2CO3, 1 mM MgSO4, and 5 mM CaCl2) in each well.

Seven *o*-cresol concentrations were used for each transfection condition in sequential dilutions of 7, alongside a control well of only reading buffer with DMSO. o-Cresol was dissolved in DMSO to 1M, and this 1M solution was diluted serially in DMSO in order to obtain six additional 7-fold dilutions. These solutions were then diluted 166.6-fold in reading buffer in order to obtain well stock solutions, and 20 µL of the well stock solution was added to each well during the imaging assay. The final o-cresol concentrations that the cells were exposed to are 1.20 mM, 171 µM, 24.5 µM 3.50 µM, 500 nM, 71.4 nM, and 10.2 nM, and all wells (including the negative control) had a concentration of .012% DMSO.

The fluorescence emission at 525 nm, with excitation at 485 nm, was continuously read by a Molecular Devices FlexStation 3 plate reader. After 16 s of baseline recording, 20 µl of odorant solution was added to the cells and read for 3 min. All plates were read at 25°C. Each concentration of ligand was applied to two technical replicates, which were averaged and considered a single biological replicate.

The baseline fluorescence (F) was calculated as the average fluorescence of the 15.4 s before o-cresol was added to the plate. Within each well, ΔF was calculated as the difference between the average of the last 15.4 s of fluorescence and the baseline F. ΔF/F was then calculated as the ΔF divided by the baseline fluorescence (F). For baseline comparisons, data collected for all mutants were normalized relative to the wild-type condition on the same plate in order to account for inevitable variations in transfection efficiency and cell counts across different plates. The normalized ΔF/F averaged across all experiments for a given condition is the value used to construct the dose–response curves in all plots. For all experiments, GraphPad Prism 10 was used to fit the dose–responses curves to the Hill equation.

Gels and small-scale transfections. For western blots and fluorescence-detection size-exclusion chromatography (FSEC) traces (Extended Data Figs. 1a, b, 9g), HEK293 cells were maintained in high-glucose DMEM supplemented with 10% (v/v) FBS and 1% (v/v) GlutaMAX at 37 °C with 5% (v/v) carbon dioxide. Cells were detached using trypsin and plated in six-well plates at a concentration of 0.4 × 106 per well. Twenty-four hours later, cells were transfected with 2 µg of DNA in the same superfolder GFP-containing pEG BacMam vector used for large-scale purification and 9 µl Lipofectamine 2000 (Invitrogen) diluted in 700 µl OptiMEM and added dropwise to the cells after a 5-min incubation. Twenty-four hours later, cells were checked for GFP fluorescence, rinsed with phosphate-buffered saline, and collected by centrifugation. Cells were either frozen at −20 °C or used immediately.

Cell pellets were rapidly thawed and resuspended in 200 µl lysis buffer containing 50 mM HEPES/NaOH (pH 7.5), 375 mM NaCl, an EDTA-free protease inhibitor cocktail (Roche), and 1 mM PMSF. The protein was extracted for 2 h at 4 °C by adding 0.5% (w/v) DDM with 0.1% (w/v) CHS after 10 s sonication in a water bath. This mixture was then clarified by centrifugation and filtered. The supernatant was added to a Shimadzu autosampler connected to a Superose 6 Increase column equilibrated in SEC buffer. An aliquot of the supernatant was also used to run SDS–PAGE (Bio-Rad, 12% Mini-PROTEAN TGX) and Blue Native(BN)-PAGE (Invitrogen, 3–12% Bis-Tris) gels. Gels were transferred using Trans-Blot Turbo Transfer Pack (Bio-Rad) and blocked overnight. The following day, gels were stained with rabbit anti-GFP polyclonal antibody (Life Technologies; 1:20,000), washed, incubated with anti-rabbit secondary antibody (1:10,000), and imaged with ImageLab.

Structure analyses. Contact residues and types of intermolecular interactions at the interfaces between subunits were determined using PDBePISA (*35*). Pore diameter along the central axis and side exits were calculated using HOLE (*62*). For each structure, separate calculations were conducted for the central pore and the side exits, varying the initial position of the probe and vector.

Sequence conservation across ORs and Orcos*.* A sequence alignment including 47 Orcos from 47 organisms as well as 461 OR sequences from 5 insect species (*An. gambiae*, *Ae. aegypti*, *D. melanogaster*, *N. vitripennis* and *P. humanus*) was constructed using MAFFT with a gap opening penalty of -3. Conservation score was calculated using JalView (*36*) based on the AMAS method of multiple sequence alignment (*63*), which measures the number of conserved physico-chemical properties conserved for each column of the alignment. Conservation is measured as a numerical index reflecting the conservation of physico-chemical properties in the alignment, with a scale that spans 1-11. Identities score highest (score = 11), and the next most conserved group (score = 10) contains substitutions to amino acids lying in the same physico-chemical class.

Figure S1.

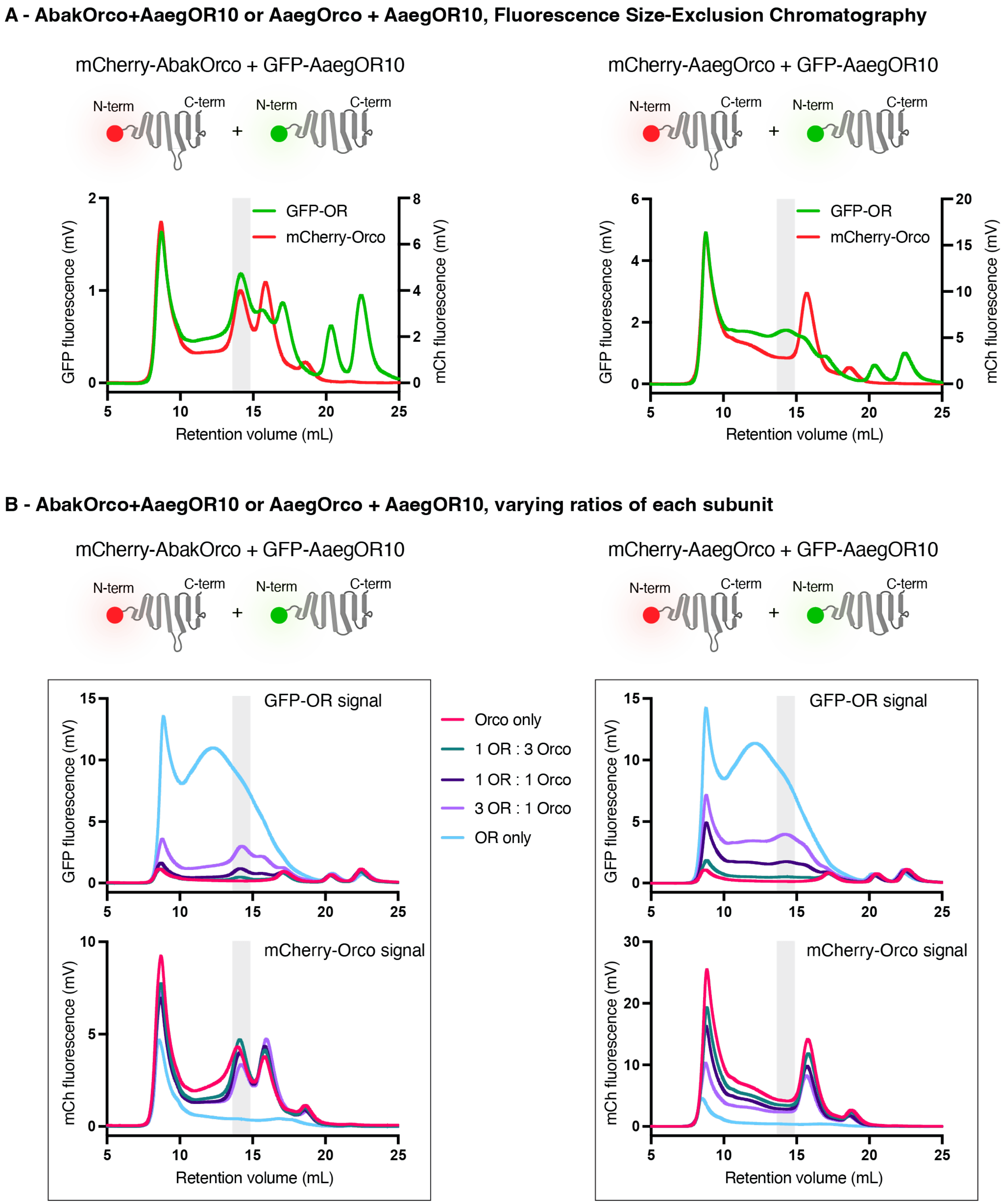

Figure S1. Biochemical behavior of *Aa. aegypti* OR10 in complex with different Orcos. (A) Fluorescence size exclusion chromatography (FSEC) profiles of solubilized HEK293 cells expressing *Aa. aegypti* OR10 in complex with *A. bakeri* Orco (left) or *Aa. aegypti* Orco (right). Complexes were expressed using transient transfection and a 1:1 ratio of DNA of each subunit.

Each protomer is tagged with either an N-terminal GFP (OR10) or an N-terminal mCherry (Orco). Gray bar marks the expected position of the heterotetramer peak, only clearly resolvable and monodisperse in the presence of *A. bakeri* Orco (left). **(B)** FSEC traces of *Aa. aegypti* OR10 in complex with *A. bakeri* Orco (left) or *Aa. aegypti* Orco (right), formed with varying ratios of OR to Orco DNA. Complexes were expressed in HEK293 cells using transient transfection and various ratios of OR to Orco DNA. Fluorescent signal of the GFP and the mCherry are depicted in separate plots within each box for clarity. All ratios containing *A. bakeri* Orco and *Aa. aegypti* OR10 exhibit a tetramer peak in the expected position (marked with a gray bar). Note that the traces shown in **(A)** are from the same experiment shown in **(B)**, corresponding to the 1:1 ratio.

Figure S2

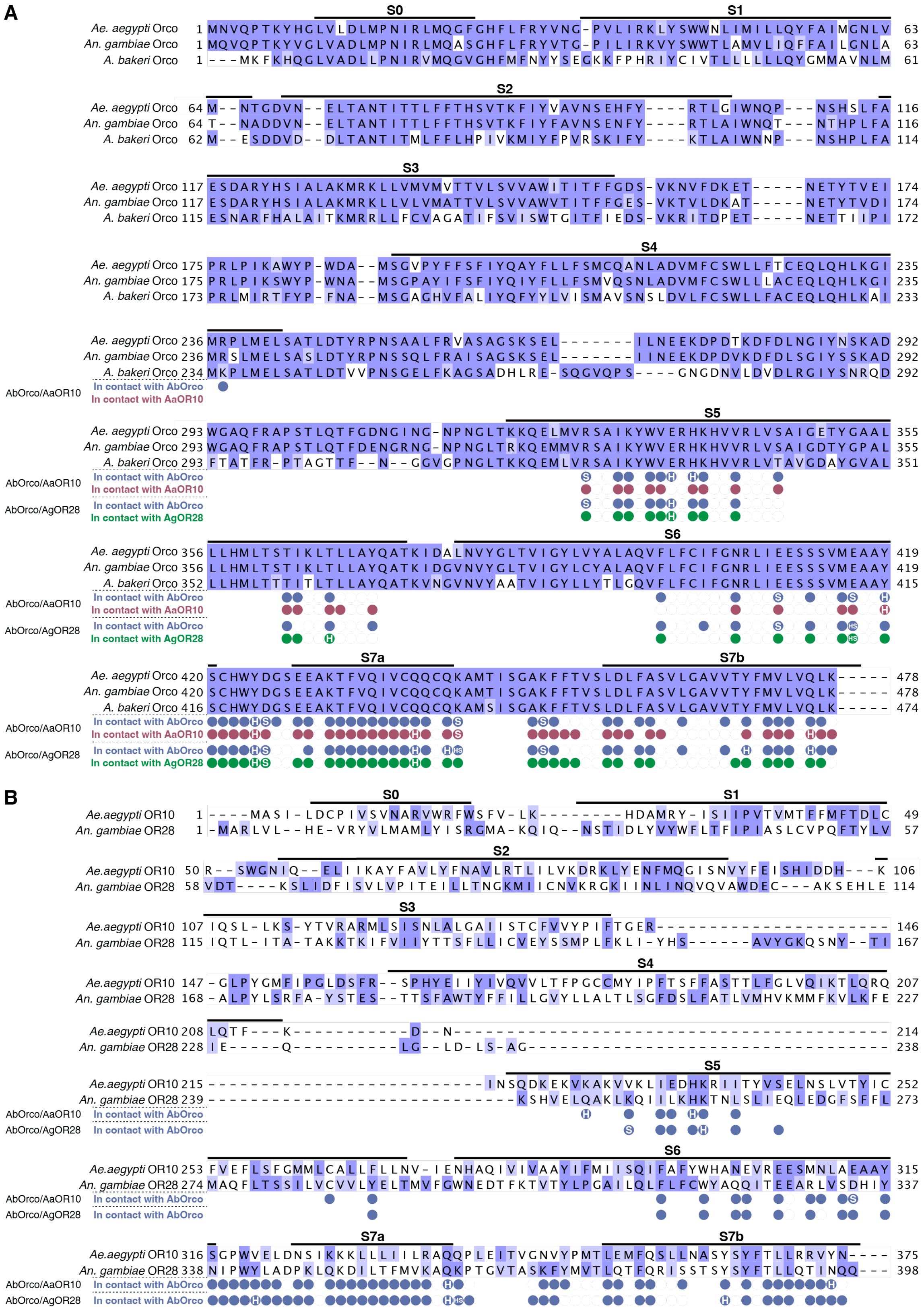

A
S0
S1
S2
S3
S4
S5
S6
S7a
S7b
Ae. aegypti Orco
An. gambiae Orco
A. bakeri Orco
AbOrco/AaOR10
AbOrco/AgOR28
In contact with AbOrco
In contact with AaOR10
In contact with AgOR28
B
Ae.aegypti OR10
An. gambiae OR28

Figure S2. Sequence alignment, conservation and position of intersubunit contact residues. Aligned amino-acid sequences of **(A)** Orcos from three species (*Ae. aegypti, An. gambiae,* and *A. bakeri*) and **(B)** the two ORs used in this study (*Ae. aegypti* OR10 and *An. gambiae* OR28). Each residue is colored by conservation from sequence alignment of 507 OR/Orco sequences. The positions of the helices observed in the structures are marked above each alignment. Contact residues and interactions, as determined by PDBePISA analysis, are noted below each alignment. Each circle underneath a particular residue denotes contacts with Orco (blue) or OR (Magenta: AaOR10, Green: AgOR28) subunits, and texts within circles indicate salt bridge interactions (S) and/or hydrogen bonding (H).

Figure S3

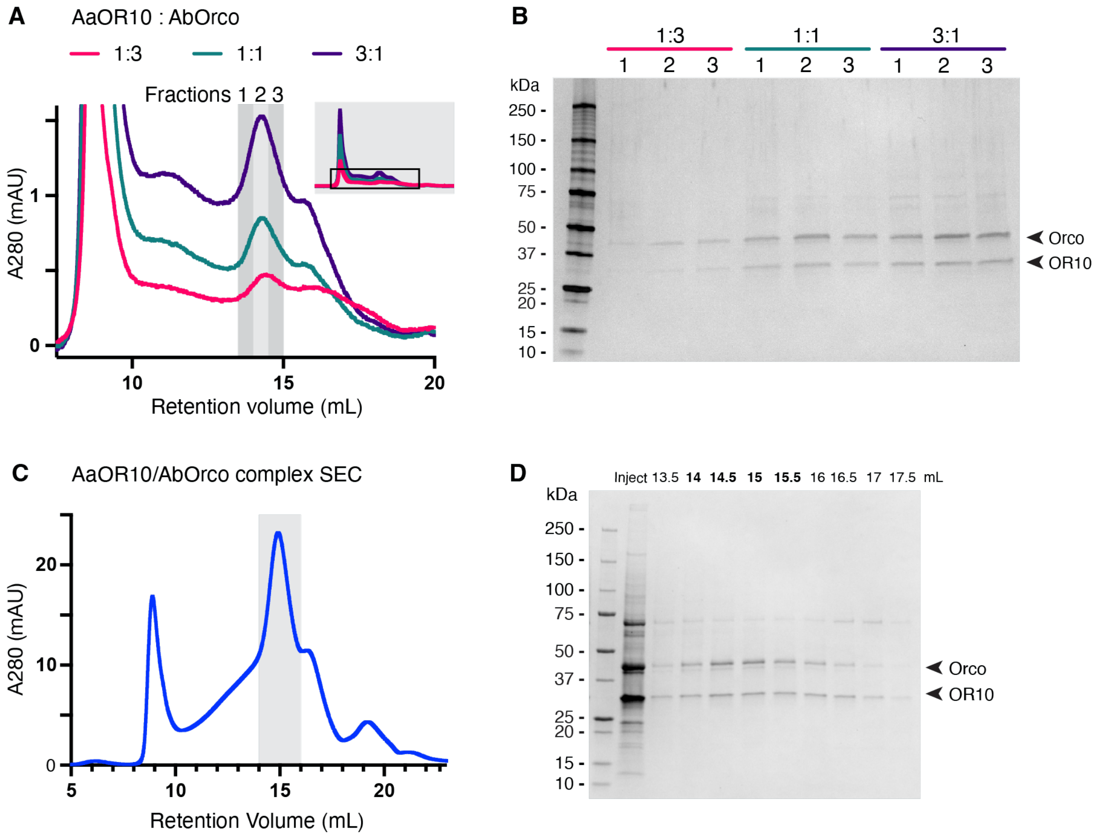

Figure S3. Purification of AaOR10/Orco. (A) Size exclusion chromatography (SEC) profile of AaOR10/AbOrco purified from HEK293 cells transfected with varying ratios or each subunit. Three fractions, each of 0.5 mL volume, were collected as highlighted in gray. Inset shows full SEC trace. **(B)** Silver-stained sodium dodecyl sulfate-poly acrylamide gel electrophoresis (SDS-PAGE) of the SEC fractions from **(A)**. Transfection ratio and fraction number are indicated above. Orco and OR10 bands are labeled with arrows. The first lane is a molecular weight ladder. **(C)** Size exclusion chromatography (SEC) profile of OR10/Orco large-scale purification used for structure determination. Fractions used for cryo-EM are highlighted in gray. **(D)** Coomassie blue-stained SDS-PAGE of SEC fractions. Inject is the sample that was injected into the SEC column.

Figure S4

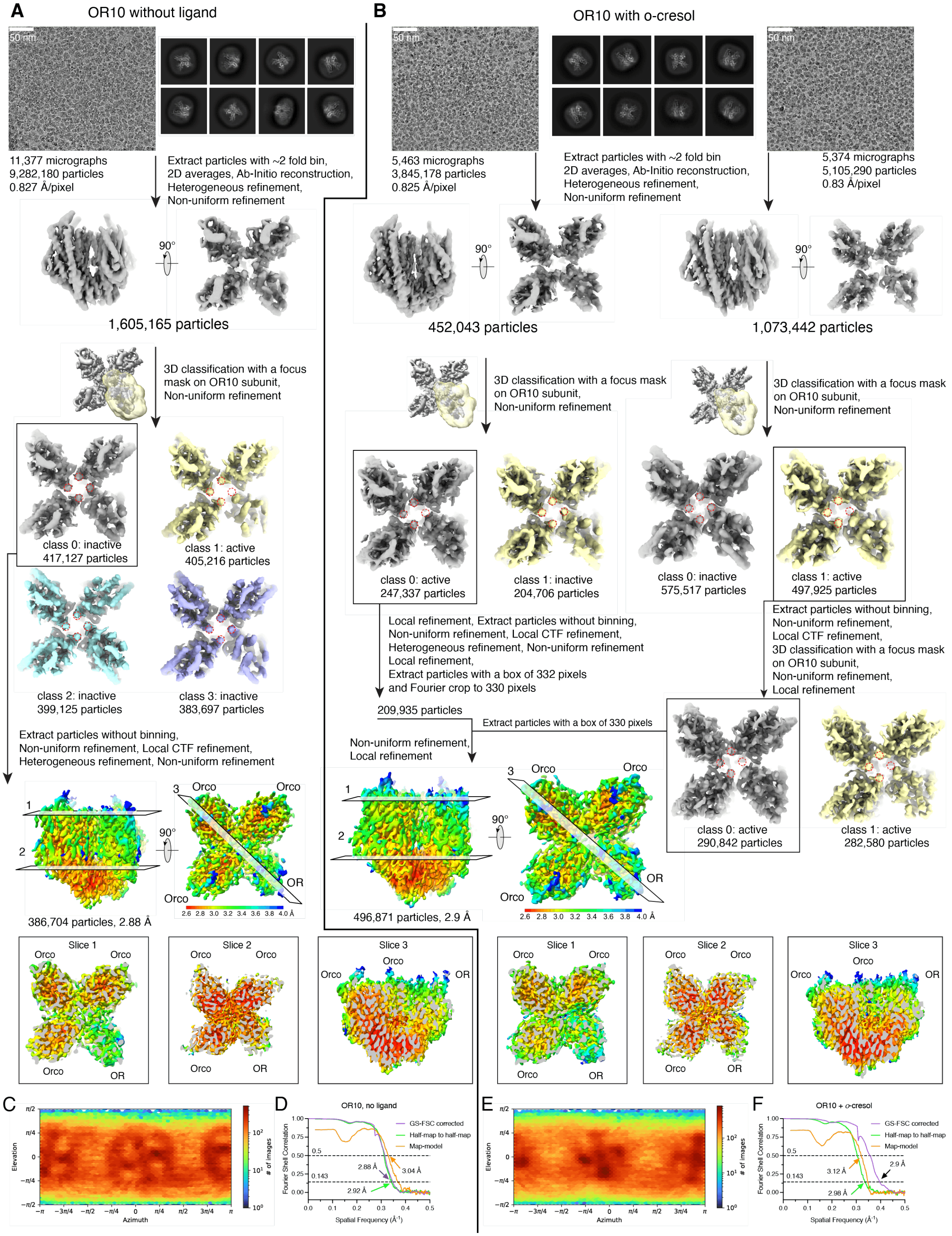

A
OR10 without ligand
50 nm
11,377 micrographs
9,282,180 particles
0.827 Å/pixel
Extract particles with ~2 fold bin,
2D averages, Ab-Initio reconstruction,
Heterogeneous refinement,
Non-uniform refinement
90°
1,605,165 particles
3D classification with a focus
mask on OR10 subunit,
Non-uniform refinement
class 0: inactive
417,127 particles
class 1: active
405,216 particles
class 2: inactive
399,125 particles
class 3: inactive
383,697 particles
Extract particles without binning,
Non-uniform refinement, Local CTF refinement,
Heterogeneous refinement, Non-uniform refinement
1
2
3
Orco
Orco
Orco
OR
90°
386,704 particles, 2.88 Å
2.6 2.8 3.0 3.2 3.4 3.6 3.8 4.0 Å
Slice 1
Slice 2
Slice 3
B
OR10 with o-cresol
50 nm
5,463 micrographs
3,845,178 particles
0.825 Å/pixel
Extract particles with ~2 fold bin
2D averages, Ab-Initio reconstruction,
Heterogeneous refinement,
Non-uniform refinement
5,374 micrographs
5,105,290 particles
0.83 Å/pixel
452,043 particles
1,073,442 particles
3D classification with a focus mask
on OR10 subunit,
Non-uniform refinement
class 0: active
247,337 particles
class 1: inactive
204,706 particles
class 0: inactive
575,517 particles
class 1: active
497,925 particles
Local refinement, Extract particles without binning,
Non-uniform refinement, Local CTF refinement,
Heterogeneous refinement, Non-uniform refinement
Local refinement,
Extract particles with a box of 332 pixels
and Fourier crop to 330 pixels
Extract particles without binning,
Non-uniform refinement,
Local CTF refinement,
3D classification with a focus mask
on OR10 subunit,
Non-uniform refinement,
Local refinement
209,935 particles
Extract particles with a box of 330 pixels
Non-uniform refinement,
Local refinement
class 0: active
290,842 particles
class 1: active
282,580 particles
496,871 particles, 2.9 Å
C
Elevation
Azimuth
# of images
D
OR10, no ligand
GS-FSC corrected
Half-map to half-map
Map-model
Fourier Shell Correlation
Spatial Frequency (Å⁻¹)
0.5
0.143
2.88 Å
3.04 Å
2.92 Å
E
F
OR10 + o-cresol
3.12 Å
2.9 Å
2.98 Å

Figure S4. Cryo-EM data processing of AaOR10/Orco. (A) and **(B)** Cryo-EM data processing of the OR10 sample without ligand (A) and OR10 with *o*-cresol (B), and the corresponding local resolution maps. The color bars indicate the local resolution of the final map. Dashed red circles indicate the S7 helices in the inactive state. **(C)** and **(E)** Angular distribution of the particles used for the final reconstructions of unbound (C) and *o*-cresol bound OR10 (E). **(D)** and **(F)**, Map and model validation for OR10 without ligand (C) and with *o*-cresol (D). Gold-standard Fourier shell correlation (GS-FSC) curves are calculated in cryoSPARC. Map-model and half-map to half-map correlations are calculated in the Phenix suite. Arrows indicate full map, half-map to half-map, and map-model resolution estimates at 0.143, 0.143, and 0.5 correlation, respectively.

Figure S5

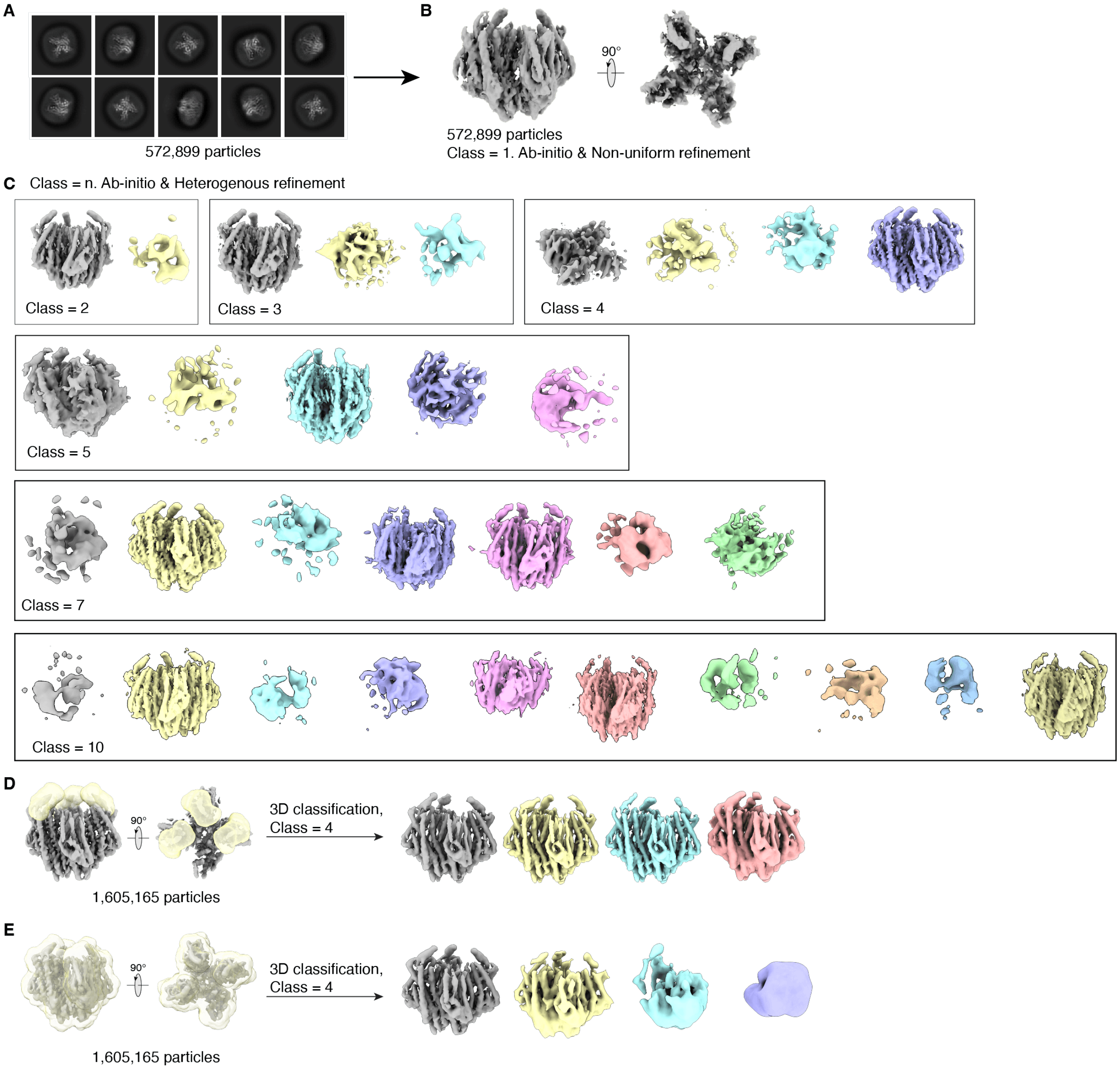

Figure S5. The AaOR10/Orco cryo-EM dataset does not contain detectable particles with other stoichiometries. (A) 2D averages of particles used for the ab-initio reconstruction and heterogenous refinement jobs. **(B)** Ab-initio reconstruction of all particles, followed by non-uniform refinement. **(C)** Ab-initio reconstruction of the same particles, requesting 2, 3, 4, 5, 7, and 10 classes in each job, followed by heterogenous refinement using references from the ab-initio reconstructions. **(D)** Focused 3D classification with a mask on the extracellular loops of Orco. **(E)** Focused 3D classification with a mask on the entire tetramer. Complete processing pipeline, including masking the OR subunit to retrieve various conformational states, can be found in fig. S4 and Methods.

Figure S6

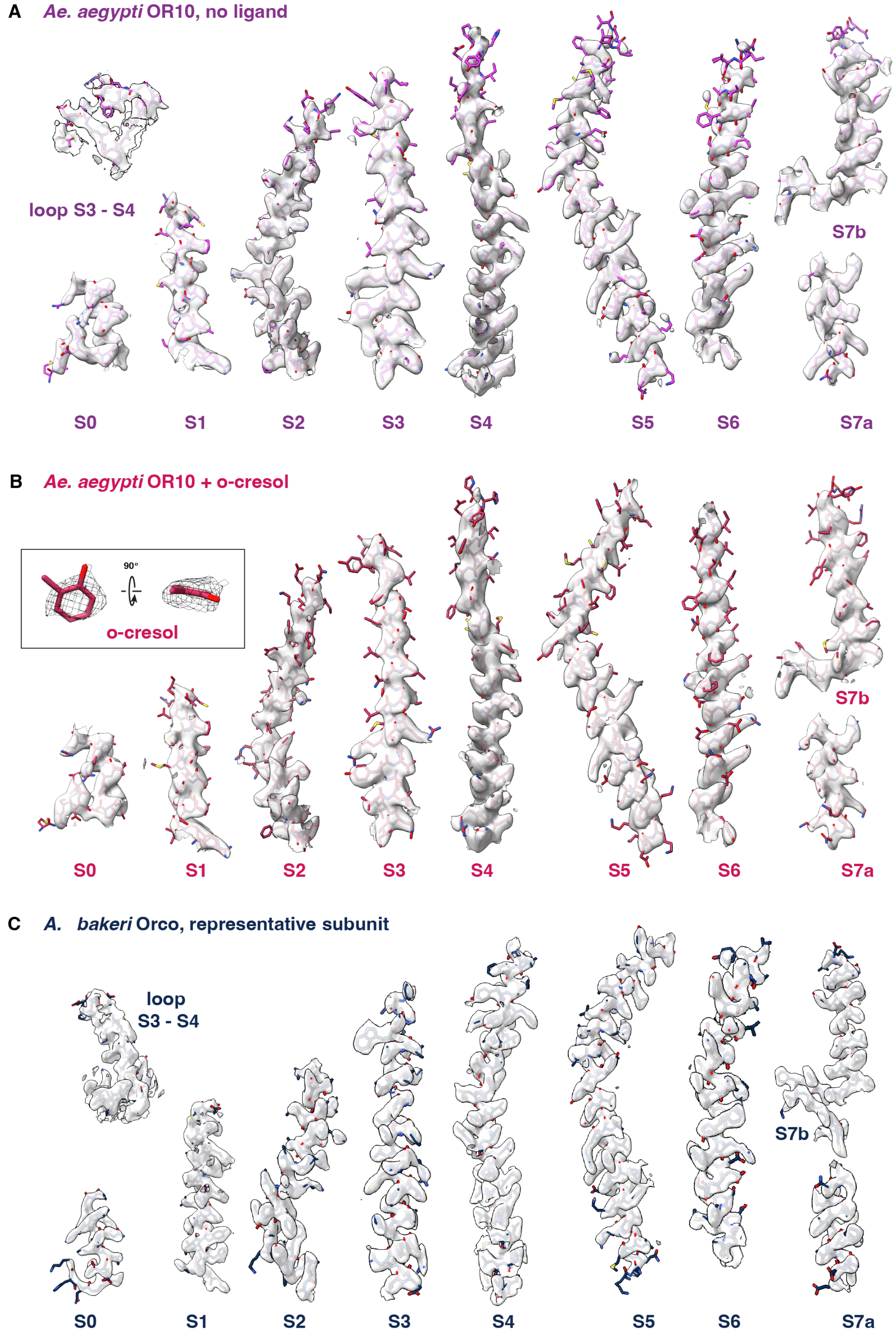
A
Ae. aegypti OR10, no ligand
loop S3 - S4
S7b
S0
S1
S2
S3
S4
S5
S6
S7a
B
Ae. aegypti OR10 + o-cresol
90°
o-cresol
S7b
S0
S1
S2
S3
S4
S5
S6
S7a
C
A. bakeri Orco, representative subunit
loop
S3 - S4
S7b
S0
S1
S2
S3
S4
S5
S6
S7a

Figure S6. Detail of the cryo-EM density of AaOR10/Orco. (A) Density for each segment of the AaOR10 subunit in the unbound state, including the extracellular S3-S4 loop that enables unambiguous subunit assignment from Orco subunits during data processing. **(B)** Density for *o*-cresol-bound AaOR10 subunit. Inset shows the density for the ligand. **(C)** Density for an Orco subunit in the unbound complex, representative of the density for all Orco subunits, which was well defined and similar for all Orco subunits.

Figure S7

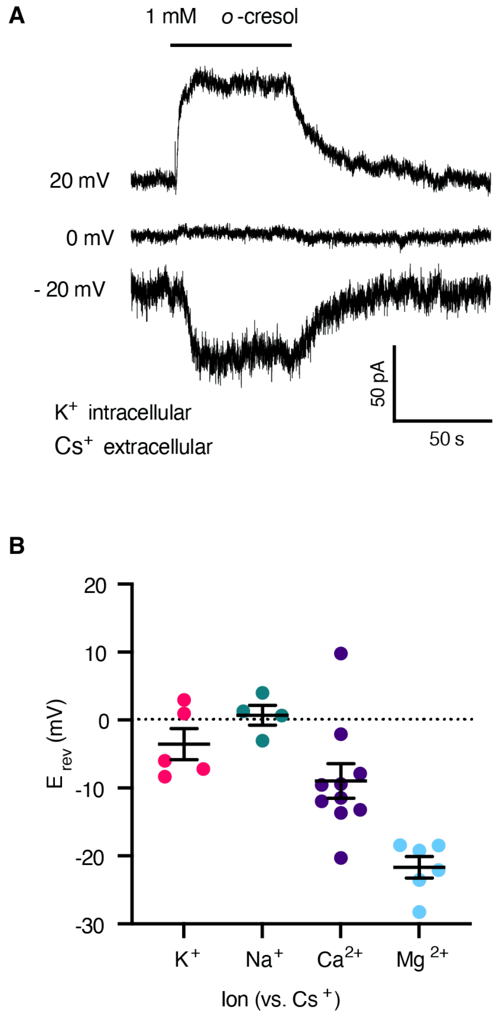

Figure S7. Ion selectivity of AaOR10/Orco complexes. (A) *o*-cresol evoked whole-cell currents from HEK293 cells expressing AaOR10+AbakOrco with 150 mM intracellular CsCl and 150 extracellular KCl, clamped at different holding voltages. **(B)** Reversal potentials (Erev; biological replicates and mean +/- SEM) for various extracellular ionic solutions *contra* 150 mM CsCl internal, determined by linear interpolation from the steady-state currents elicited by *o*-cresol at holding potentials around the reversal, and corrected for liquid junction potentials. Also see data table S3.

Figure S8

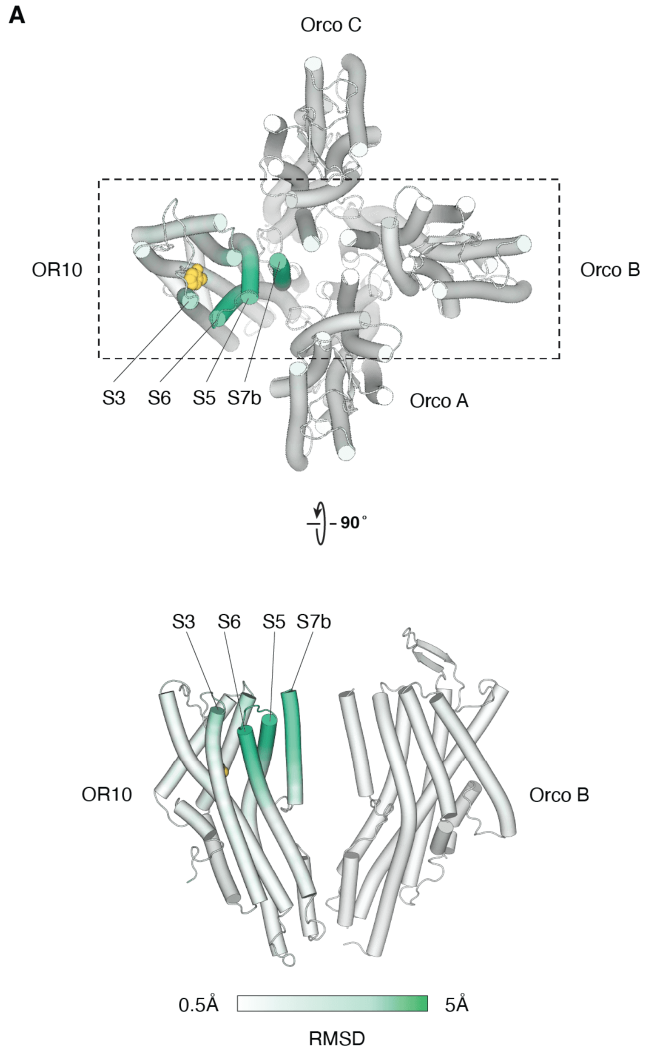

Figure S8. RMSD between the unbound and odorant-bound models of the AaOR10/Orco complexes. (A) Top view of the *o*-cresol bound model, colored by RMSD calculated between the odorant-bound and unbound structures. Bottom: side view of the OR and the diagonally opposing Orco subunit. Only the OR subunit experiences significant rearrangements between both conformations. The position of relevant helices experiencing largest conformational changes is shown (S7, S5, S6 and S3).

Figure S9

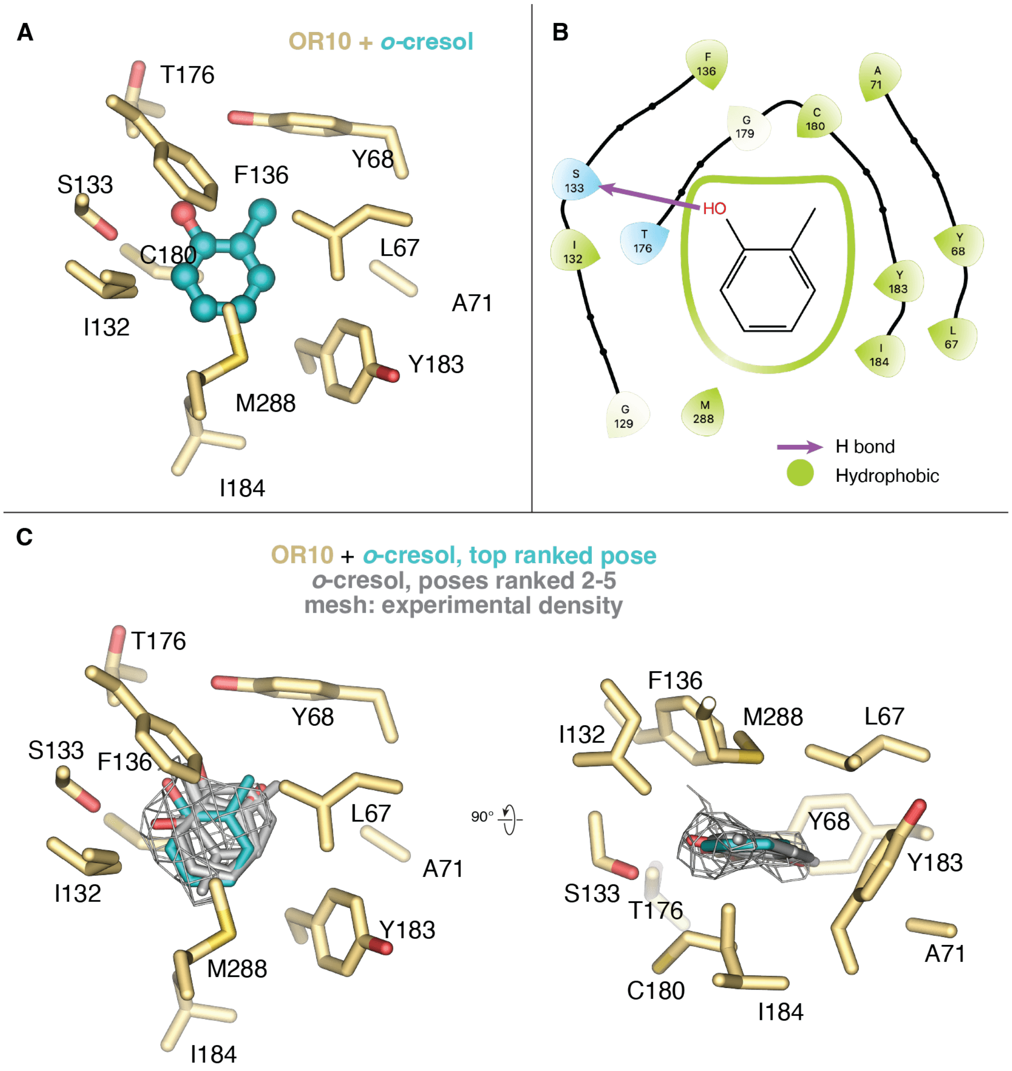

Figure S9. Details of the AaOR10/Orco odorant binding pocket and binding mode of *o*-cresol. (A) Detail of the *o*-cresol-bound structure, depicting the residues involved in ligand binding according to the ligand-interaction analysis conducted Maestro (Schrödinger). **(B)** 2D ligand-interaction plot, showing the interactions mediating *o*-cresol binding. The majority of the interactions are hydrophobic, and a hydrogen bond is established between the hydroxyl group of the ligand and serine 133. **(C)** Left, same view of the binding pocket as in **(A)**, showing the EM density for the ligand (gray mesh) and the top 5 binding poses obtained by docking the ligand into the binding pocket using Glide (Schrödinger). The top binding pose was used for modeling the ligand and is depicted in cyan, the other 4 poses are shown in gray. All top binding poses fit well within the experimental density with docking scores: -6.794, -6.441, -6.409, -6.254, -6.169. Right, rotated view of the pocket with same elements as in **(C)**.

Figure S10

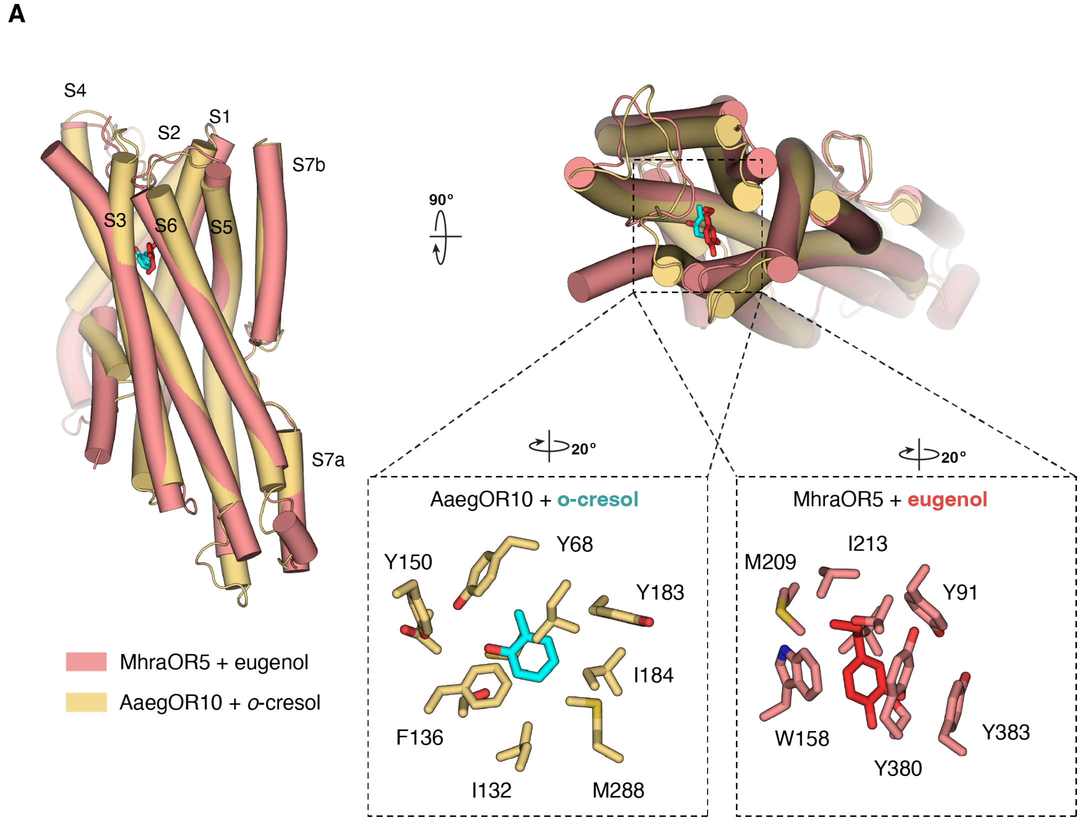

Figure S10. Comparison of odorant-binding pockets of insect odorant receptors structurally characterized to date. (A) Overlay of the cryo-EM structures of AaOR10 bound to *o*-cresol and MhOR5 bound to eugenol, in lateral and top views. Insets detail the residues interacting with the respective odorant ligands.

Figure S11

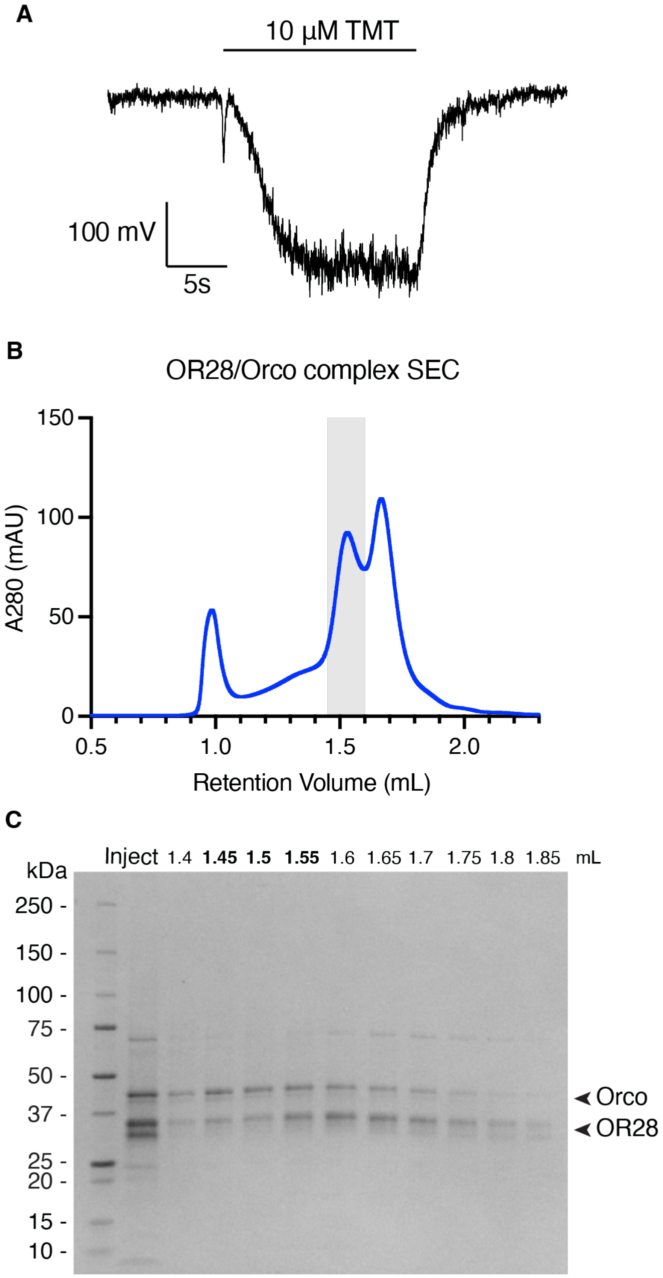

Figure S11. Functional characterization and purification of AgOR28/Orco complex. (A) representative whole-cell current evoked by 2,4,5-trimethylthiazole (TMT) application in a HEK293 cell expressing AgOR28/Orco, voltage clamped at -80 mV. **(B)** Size exclusion chromatography (SEC) profile of OR28/Orco purification from HEK293 cells. Fractions used for cryo-EM are highlighted in grey. **(C)** Sodium dodecyl sulfate-polyacrylamide gel electrophoresis (SDS-PAGE) of SEC fractions. The first lane is a molecular weight ladder. Inject, the sample that was injected into the SEC column. The numbers on top indicate the retention volume in **(A)**. Fractions used for cryo-EM are highlighted in bold. Orco and OR28 bands are labeled with arrows.

Figure S12

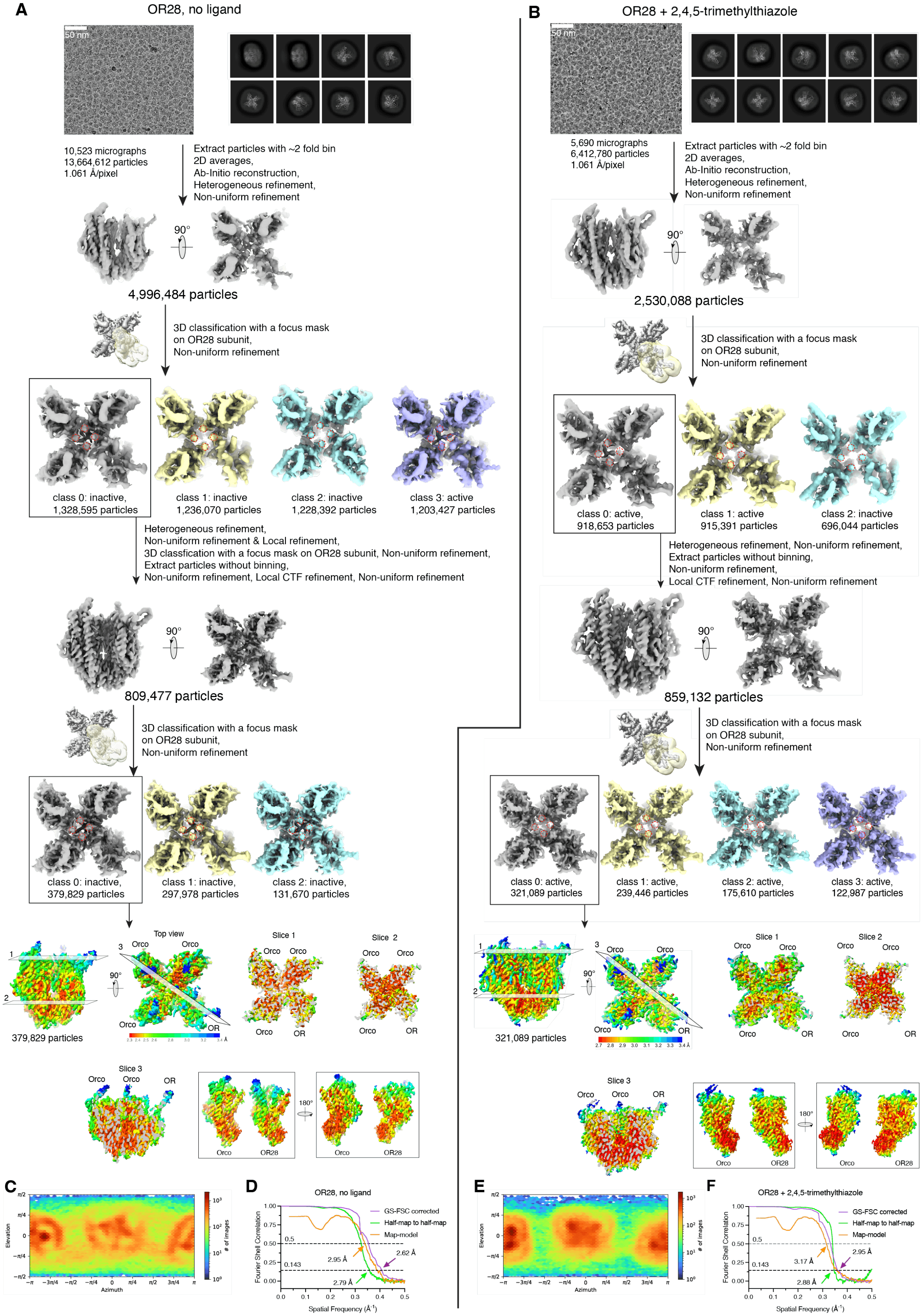
A
OR28, no ligand
50 nm
10,523 micrographs
13,664,612 particles
1.061 Å/pixel
Extract particles with ~2 fold bin
2D averages,
Ab-Initio reconstruction,
Heterogeneous refinement,
Non-uniform refinement
90°
4,996,484 particles
3D classification with a focus mask
on OR28 subunit,
Non-uniform refinement
class 0: inactive,
1,328,595 particles
class 1: inactive
1,236,070 particles
class 2: inactive
1,228,392 particles
class 3: active
1,203,427 particles
Heterogeneous refinement,
Non-uniform refinement & Local refinement,
3D classfication with a focus mask on OR28 subunit, Non-uniform refinement,
Extract particles without binning,
Non-uniform refinement, Local CTF refinement, Non-uniform refinement
90°
809,477 particles
3D classification with a focus mask
on OR28 subunit,
Non-uniform refinement
class 0: inactive,
379,829 particles
class 1: inactive,
297,978 particles
class 2: inactive,
131,670 particles
Top view
Slice 1
Slice 2
Orco
OR
379,829 particles
Slice 3
180°
OR28
B
OR28 + 2,4,5-trimethylthiazole
50 nm
5,690 micrographs
6,412,780 particles
1.061 Å/pixel
Extract particles with ~2 fold bin
2D averages,
Ab-Initio reconstruction,
Heterogeneous refinement,
Non-uniform refinement
90°
2,530,088 particles
3D classification with a focus mask
on OR28 subunit,
Non-uniform refinement
class 0: active,
918,653 particles
class 1: active
915,391 particles
class 2: inactive
696,044 particles
Heterogeneous refinement, Non-uniform refinement,
Extract particles without binning,
Non-uniform refinement,
Local CTF refinement, Non-uniform refinement
90°
859,132 particles
3D classification with a focus mask
on OR28 subunit,
Non-uniform refinement
class 0: active,
321,089 particles
class 1: active,
239,446 particles
class 2: active,
175,610 particles
class 3: active,
122,987 particles
321,089 particles
C
Elevation
Azimuth
# of images
D
OR28, no ligand
GS-FSC corrected
Half-map to half-map
Map-model
Fourier Shell Correlation
Spatial Frequency (Å⁻¹)
0.5
0.143
2.95 Å
2.62 Å
2.79 Å
E
F
OR28 + 2,4,5-trimethylthiazole
3.17 Å
2.95 Å
2.88 Å

Figure S12. Cryo-EM data analysis of the AgOR28/Orco complex. (A) and **(B)** Cryo-EM data processing of the OR28 sample without ligand (A) and OR28with 2,4,5-trimethylthiazole (TMT) (B), and the corresponding local resolution maps. The color bars indicate the local resolution of the final map. Dashed red circles indicate the S7 helices in the inactive state. **(C) and (E)** Angular distribution of the particles used for the final reconstructions of unbound (C) and TMT bound OR28 (E). **(D)** and **(F)**, Map and model validation for OR28 without ligand (C) and with TMT (D). Gold-standard Fourier shell correlation (GS-FSC) curves are calculated in cryoSPARC. Map-model and half-map to half-map correlations are calculated in the Phenix suite. Arrows indicate full map, half-map to half-map, and map-model resolution estimates at 0.143, 0.143, and 0.5 correlation, respectively.

Figure S13

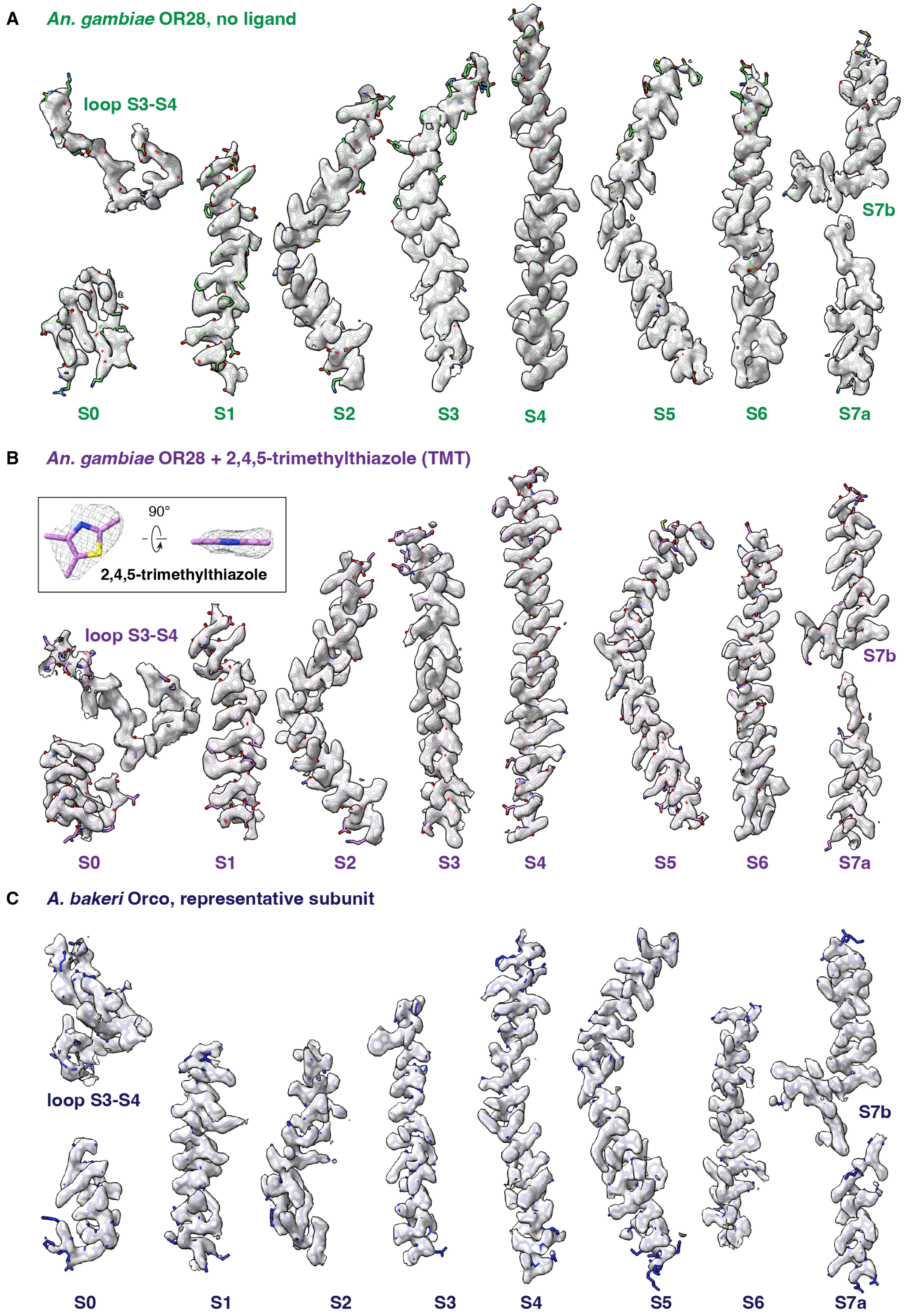
A
An. gambiae OR28, no ligand
loop S3-S4
S7b
S0
S1
S2
S3
S4
S5
S6
S7a
B
An. gambiae OR28 + 2,4,5-trimethylthiazole (TMT)
90°
2,4,5-trimethylthiazole
loop S3-S4
S7b
S0
S1
S2
S3
S4
S5
S6
S7a
C
A. bakeri Orco, representative subunit
loop S3-S4
S7b
S0
S1
S2
S3
S4
S5
S6
S7a

Figure S13. Detail of the cryo-EM density of AgOR28/Orco. (A) Density for each segment of the AgOR28 subunit in the unbound state, including the extracellular S3-S4 loop that enables unambiguous subunit assignment from Orco subunits during data processing. **(B)** Density for 2,4,5-trimethylthiazole-bound AgOR28 subunit. Inset shows the density for the ligand. **(C)** Density for an Orco subunit in the unbound complex, representative of the density for all Orco subunits, which was well defined and similar for all Orco subunits.

Figure S14

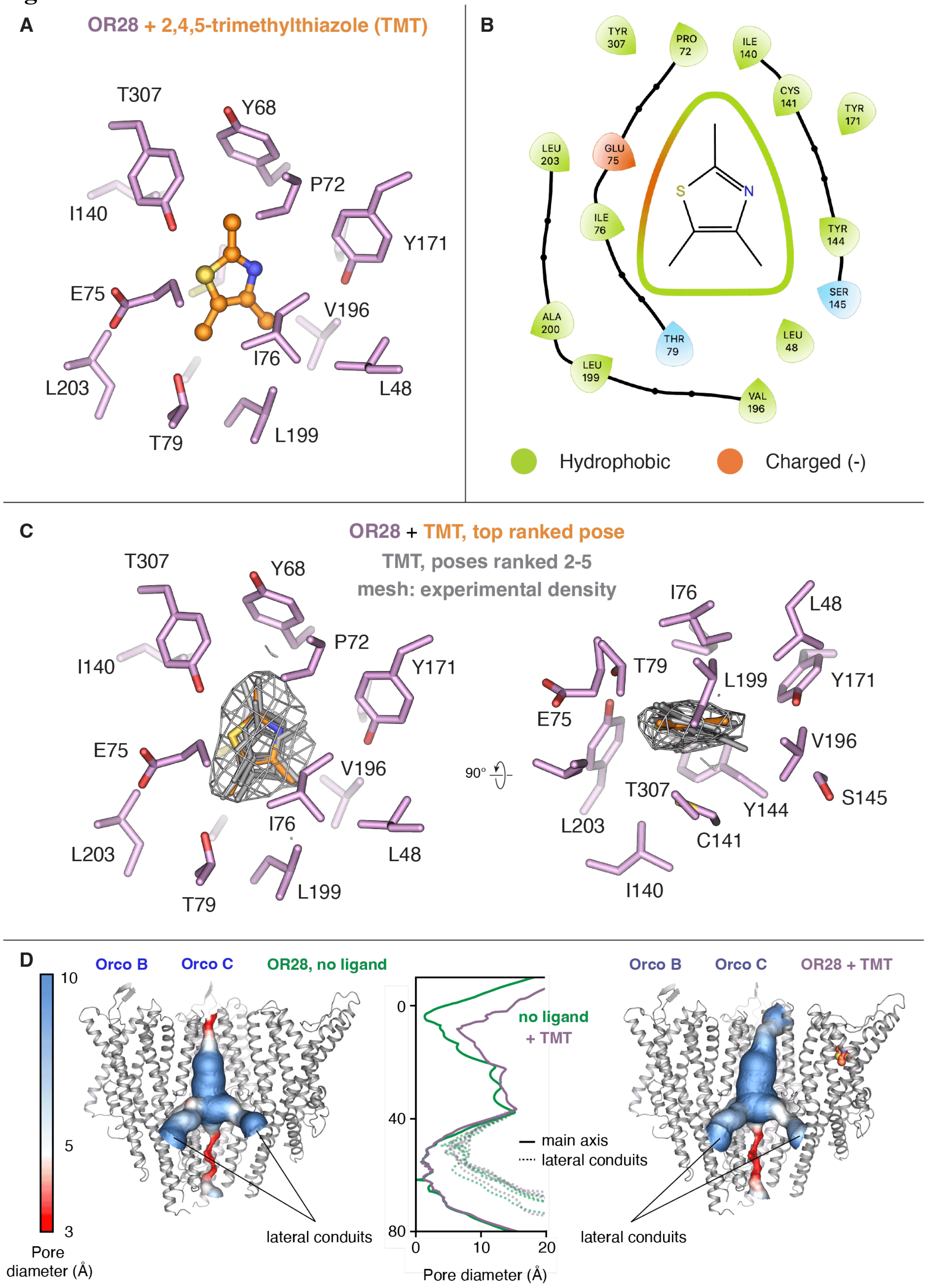

Figure S14. Details of the odorant binding pocket, binding mode of 2,4,5-trimethylthiazole (TMT) and details of the pore opening of AgOR28/Orco. (A) Detail of the TMT-bound structure, depicting the residues involved in ligand binding according to the ligand-interaction analysis conducted Maestro (Schrödinger). **(B)** 2D ligand-interaction plot, showing the

interactions mediating TMT binding. Except for a polar interaction with the charged side chain of Glu 75, the majority of the interactions are hydrophobic. **(C)** Left, same view of the binding pocket as in **(A)**, showing the EM density for the ligand (gray mesh) and the top 5 binding poses obtained by docking the ligand into the binding pocket using Glide (Schrödinger). The top binding pose was used for modeling the ligand and is depicted in orange, the other 4 poses are shown in gray. All top binding poses fit well within the experimental density with docking scores: -5.944, -5.937, -5.934, -5.834, -5.783. Right, rotated view of the pocket with same elements as in **(C)**. **(D)** The ion permeation pathways of the unbound (left) and TMT-bound (right) structures, colored by pore diameter. The front Orco A subunit is not shown to permit visualization of the cavity. The center vestibule of the ion conduction pathway is continuous with four lateral conduits that allow ion permeation; two are shown in each structure. The intracellular 'anchor' domain remains closed in unbound and TMT-bound structures. The plot shows the diameter of the ion permeation pathway, with respect to distance from the outer membrane boundary towards the intracellular space, in Å. The diameter of the impermeable central path through the anchor domain is shown in solid lines, while those of the lateral conduits are in dashed lines.

Table S1. Cryo-EM data collection, refinement and model validation statistics.

	AaegOR10/AbakOrco apo	AaegOR10/AbakOrco *o*-cresol	AgamOR28/AbakOrco apo	AgamOR28/AbakOrco 2,4,5-tmt
PDB	8V00	8V02	8V3C	8V3D
EMDB	EMD-42848	EMD-42850	EMD-42945	EMD-42946
**Data collection and Processing**	-	-	-	-
Microscope	Titan Krios			
Voltage (kV)	300			
Camera	Gatan K3			
Magnification	105,000		81,000	
Pixel size at detector (Å /pixel)	0.827	0.825 and 0.83	1.061	1.061
Total Electron exposure ($e^-/Å^2$)	60	55 and 58	50	50
Number of frames collected during exposure	60	58 and 64	50	50
Defocus range (µm)	-1 to -2	-1 to -2	-1 to -2	-1 to -2
Automation software	SerialEM			
Tilt angle	0 and 30°			
Micrographs used	11,377	10,837	1,0523	5,690
Total extracted particles	9,282,180	8,950,468	13,664,612	6,412,780
**For each reconstruction:**	-	-	-	-
Final particles	386,704	496,871	377,441	327,342

Symmetry imposed	C1	C1	C1	C1
Map sharpening B factor (Å^2)	-100	-91.8	-50	-100.9
Map resolution (Å)	2.88	2.90	2.62	2.95
Map resolution range (Å)	1.775 - 37.472	1.797 - 34.846	2.342 - 29.704	2.519 - 40.474
FSC threshold	0.143			
**Model refinement**	-	-		-
Non-hydrogen atoms	12665	12442	12613	12636
Protein residues	15577	1548	1576	1578
Protein B factor (Å^2)	55.99	37.87	80.37	34.95
Ligand B factor (Å^2)	n.a.	68.28	n.a.	29.78
Bond length RMSD (Å)	0.004	0.003	0.004	0.003
Bond angle RMSD (°)	0.573	0.549	0.535	0.494
Ramachadran favored (%)	97.07	96.40	97.49	96.98
Ramachadran allowed (%)	2.93	3.60	2.51	3.02
Ramachadran outliers (%)	0.00	0.00	0.00	0.00
Rotamer outliers (%)	5.11	3.60	3.38	2.66
MolProbity score	2.29	2.27	1.96	1.84

MolProbity clash score	11.74	12.62	8.32	6.13

Table S2. OR10 electrophysiology dose-response parameters and sample sizes.

	Ligand	N (cells)	N (samples)	Mean EC50 (µM)	Log EC50 ± SE	Hill Slope ± SE	p value
AaegOR10 /AaegOrco	indole	4	7	1.51	-5.821± 0.04875	0.9132 ± 0.08519	0.1363
AaegOR10 /AbakOrco	indole	4	7	1.091	-5.962 ± 0.04781	0.9628 ± 0.09928	-
AaegOR10 /AaegOrco	*o*-cresol	4	7	11.32	-4.946 ± 0.1473	0.6854 ± 0.1796	0.9334
AaegOR10 /AbakOrco	*o*-cresol	3	5	13.31	-4.876 ± 0.1191	0.6839 ± 0.1428	-

Table S3. Reversal potentials for ion selectivity studies for OR10/AbakOrco stimulated with *o*-cresol.

Ion	N	E_{rev} Mean ± SD (mV)
Ca^{2+}	10	-9.071 ± 8.055
Mg^{2+}	6	-21.76 ± 3.86
K^{+}	5	-3.630 ± 5.124
Na^{+}	4	0.6212 ± 2.913

Table S4. OR10 mutant dose-response parameters and baselines. Only conditions in which there are at least two points around saturation have EC50 reported.

Mutant	N	Mean EC50 (uM)	Log EC50 ± SE	Hill Slope ± SE	Baseline (normalized) ± SE	p value (Baseline)
WT	7	7.907	-5.102 ± 0.1319	0.7766 ± 0.1717	-	

S133A	6	-	-	-	0.9064 ± 0.01433	0.0013
L67A	6	-	-	-	0.9298 ± 0.03249	0.083
Y183A	6	-	-	-	0.9250 ± 0.04652	0.1678
F136A	6	9.843	-5.007 ± 0.1691	0.7524 ± 0.2069	1.170 ± 0.06277	0.0426
N125A	6	-	-	-	0.8882 ± 0.03848	0.0336
Q292A	5	-	-	-	0.8904 ± 0.03043	0.0227
